# Taurine-mediated gene transcription and cell membrane permeability reinforced co-production of bioethanol and Monascus azaphilone pigments for a newly isolated *Monascus purpureus*

**DOI:** 10.1186/s13068-024-02511-7

**Published:** 2024-05-03

**Authors:** Xia Yi, Jianqi Han, Xiaoyan Xu, Yilong Wang, Meng Zhang, Jie Zhu, Yucai He

**Affiliations:** 1 National-Local Joint Engineering Research Center for Biomass Refining and High-Quality Utilization, Institute of Urban and Rural Mining, Changzhou Key Laboratory of Biomass Green, Safe & High Value Utilization Technology, School of Petrochemical Engineering, Changzhou University, Changzhou, 213164 Jiangsu China; 2 School of Pharmacy & School of Biological and Food Engineering, Changzhou University, Changzhou, 213164 Jiangsu China

**Keywords:** *Monascus purpureus*, Bioethanol, MonAzPs, Taurine, Cell membrane permeability

## Abstract

**Background:**

Taurine, a semi-essential micronutrient, could be utilized as a sulfur source for some bacteria; however, little is known about its effect on the accumulation of fermentation products. Here, it investigated the effect of taurine on co-production of bioethanol and Monascus azaphilone pigments (MonAzPs) for a fungus.

**Results:**

A newly isolated fungus of 98.92% identity with *Monascus purpureus* co-produced 23.43 g/L bioethanol and 66.12, 78.01 and 62.37 U/mL red, yellow and orange MonAzPs for 3 d in synthetic medium (SM). Taurine enhanced bioethanol titer, ethanol productivity and ethanol yield at the maximum by 1.56, 1.58 and 1.60 times than those of the control in corn stover hydrolysates (CSH), and red, yellow and orange MonAzPs were raised by 1.24, 1.26 and 1.29 times, respectively. Taurine was consumed extremely small quantities for *M. purpureus* and its promotional effect was not universal for the other two biorefinery fermenting strains. Taurine intensified the gene transcription of glycolysis (glucokinase, phosphoglycerate mutase, enolase and alcohol dehydrogenase) and MonAzPs biosynthesis (serine hydrolases, C-11-ketoreductase, FAD-dependent monooxygenase, 4-*O*-acyltransferase, deacetylase, NAD(P)H-dependent oxidoredutase, FAD-dependent oxidoredutase, enoyl reductase and fatty acid synthase) through de novo RNA-Seq assays. Furthermore, taurine improved cell membrane permeability through changing cell membrane structure by microscopic imaging assays.

**Conclusions:**

Taurine reinforced co-production of bioethanol and MonAzPs by increasing gene transcription level and cell membrane permeability for *M. purpureus*. This work would offer an innovative, efficient and taurine-based co-production system for mass accumulation of the value-added biofuels and biochemicals from lignocellulosic biomass.

**Supplementary Information:**

The online version contains supplementary material available at 10.1186/s13068-024-02511-7.

## Background

For the desirable characteristics of ideal octane value and combustion efficiency, bioethanol is regarded as one of the most promising alternatives to the conventional transport fuels in the future [1]. The use of bioethanol as transport fuel will really reduce the buildup of carbon dioxide. Nearly approaching carbon neutral, lignocellulosic biomass available in massive quantities can be widely used to produce bioethanol [2]. Therefore, an increasing focus is on the acquirement of the robust strains to augment bioethanol production in biorefinery fields [3]. The classic producers such as *Saccharomyces cerevisiae* and *Zymomonas mobilis* are used for bioethanol fermentation with pure sugar and various lignocellulosic biomass [4, 5]. However, little was known on bioethanol production of *Monascus purpureus*.

The filamentous fungus *M. purpureus*, known for red yeast rice fermentation [6] and wine starters of brewage industry [7], has been widely used as edible pigments [8], polysaccharides [9] and medicinal agents [10]*.* Monascus pigments, more precisely, Monascus azaphilone pigments (MonAzPs), are a kind of complex compound mixtures including red (rubropunctamine and monascorubramine), orange (rubropunctatin and monascorubrin) and yellow (monascin and ankaflavin) pigments shared the common skeleton of azaphilone [11]. MonAzPs are widely used as food colorants [12], pharmaceutical [13] and textile dyeing industries [14]. Therefore, a lot of efforts including fermentation process optimization [15] and the operation of genetic engineering [9], metabolic engineering [16, 17] and systems biology [18], have been made to improve MonAzPs productivity. Biomass substrates are also used to produce MonAzPs from *M. purpureus* [19]. Exogenous amino acids, such as S-adenosylmethionine (SAM), histidine and methionine, are increasingly standing out for their advantages of time saving, low cost and simple operation to facilitate MonAzPs biosynthesis [20, 21]. However, little was known for co-production of bioethanol and MonAzPs from CSH for *M. purpureus* treated with taurine.

Taurine (2-aminoethanesulfonic acid), the main end-product of cysteine metabolism in eukaryotes, can be synthesized through metabolic engineering and chemical synthesis [22]. As a semi-essential micro-nutrient, taurine was always biologically and physiologically used in food, energy drinks and medicine [22–24]. Although used as a sulfur source for fermenting bacteria [25], the effect of taurine on product accumulation of *M. purpureus* is unknown.

Co-production systems were considered to be an ideal biorefinery strategy [26]. Here, it firstly investigated co-production ability of bioethanol and MonAzPs for a newly isolated fungus. It further assayed the effect of taurine on co-production of bioethanol and MonAzPs from synthetic medium (SM) and corn stover hydrolysates (CSH). Additionally, it also assessed the universality of the promotional effects for taurine on the two classic biorefinery strains. Further, deep sequencing assays were carried out to uncover gene transcriptional change in *M. purpureus* treated with taurine. The morphology and structure of *M. purpureus* treated with taurine were also studied using microscopic imaging assays. This study would offer a taurine-based efficient co-production system for mass accumulation of the value-added biofuels and biochemicals from lignocellulosic biomass.

## Materials and methods

### Reagents

The commercial cellulase was purchased from Sigma-Aldrich (St. Louis, MO, USA) and the filter paper activity was determined as the document [5]. Taurine (HPLC ≥ 98%) was from Yuanye Biotechnology Co., Ltd (Shanghai, China). Propidium iodide (PI) was purchased from Shanghai Macklin Biochemical Technology Co., Ltd. All the other analytical grade chemicals were purchased from China National Pharmaceutical Group Co., Ltd (Sinopharm).

### Strain identification and culture

*M. purpureus* MP2022 (CGMCC3.25392) was isolated from the wet rubbish soil of Yancheng Relics Park in Changzhou, Jiangsu province of China. PCR-sequencing of ITS1 (internal transcribed spacer)–5.8 S rDNA–ITS4 region was carried out by Jiangsu Genecefe Biotechnology Co., Ltd (Wuxi, China). MEGA (Version 6.0) software package with neighbor-joining method was used to construct the phylogenetic tree.

The seed slant of *M. purpureus* was prepared at 30 °C for 4 d on potato-dextrose agar (PDA) medium containing 200.0 g/L potato, 20.0 g/L glucose and 15.0 g/L agar. Rinsed from a seed slant with 5.0 mL liquid PDA medium, a 2.0-mL seed slant cultures (approximately 4.0 × l0^6^ spores/mL) was cultured at 30 °C for 1 d in 50 mL seed activated medium containing 20.0 g/L glucose, 3.0 g/L peptone, 4.0 g/L yeast, 20.0 g/L malt, 2.0 g/L KH_2_PO_4_, 2.0 g/L NaNO_3_ and 1.0 g/L MgSO_4_·7H_2_O in 250-mL Erlenmeyer flask with slight modification [27]. A 5.0 mL activated cultures was further inoculated in 50 mL seed medium at 30 °C and 200 rpm for 1 d [27]. A 5.0 mL seed cultures was inoculated in 50 mL fermentation medium at 25 °C and 150 rpm for 4 d after pre-cultured at 30 °C and 150 rpm for 2 d [27]. Sampling was at 1 d interval. Taurine with the final concentration of 2.0 g/L, 4.0 g/L, 6.0 g/L, 8.0 g/L and 10.0 g/L was separately amended in fermentation medium sterilized at 121 °C for 15 min, and no taurine was added for the control. For lignocellulosic biomass fermentation, a 5.0-mL seed culture was inoculated in the two following media: (1) CSH system was amended with 4.0 g/L taurine, and no taurine was added for the control; and (2) CSH system was simultaneously amended with 4.0 g/L taurine and the fermentation medium nutrients other than glucose, and no taurine was added for the control. For the assays of de novo RNA-Seq and microscopic assays, the fresh mycelia of *M. purpureus* treated with 4.0 g/L taurine in fermentation medium for 1 d were harvested from a 50 mL fermentation medium, and no taurine was added for the control. All assays were carried out in triplicate.

The ethanologenic bacterium *Z. mobilis* ZM4 (ATCC 31821) was cultured in RM (Rich Medium) medium [5]. A 10 mL RM culture was inoculated in 100 mL RM medium amended with the final concentration of 2.0, 4.0, 6.0 and 8.0 g/L taurine in 250-mL Erlenmeyer flask at 30 °C without shaking. Sampling was at 4-h interval. All assays were carried out in triplicate.

The itaconic acid-producing fungus *Aspergillus terreus* AT2022 (CGMCC3.25393) was prepared in PDA slant medium. Seed medium and fermentation medium were prepared according to the previous method with slight modification by replacing ammonium nitrate with ammonium sulfate [28]. After rinsing a slant with a 5.0 mL liquid PDA medium, a 2.0-mL spore suspension (approximately 5.0 × l0^6^ spores/mL) was inoculated in 50 mL seed medium with 250-mL Erlenmeyer flasks and cultured at 32 °C and 180 rpm for 1 d. A 5.0-mL seed culture was cultured at 32 °C for 8 d in 50 mL fermentation medium amended with the final concentration of 1.4, 2.8, 4.2 and 5.6 g/L taurine. Sampling was at 2 d interval. All assays were carried out in triplicate.

### CSH pretreatment

Corn stover was pretreated according to the previous study [5]. Enzymatic saccharification assays were carried out at 48 °C for 3 d. CSH contained 46.24 g/L glucose, 16.53 g/L xylose, 0.12 g/L furfural, 0.83 g/L HMF, 0.01 g/L 4-hydroxybenzaldehyde, 0.31 g/L syringaldehyde and 0.09 g/L vanillin.

### De novo RNA-Seq

To uncover the molecular mechanism of the promotional effect of taurine on co-production of bioethanol and MonAzPs, de novo transcriptomic sequencing assays were carried out for *M. purpureus* by BGI Genomics Co., Ltd (Shenzhen, China). The total RNA was isolated from *M. purpureus* using TRlzol Reagent following the commercial instructions (Life technologies, California, USA). The differentially expressed genes (DEGs) were defined as the absolute value of foldchange ≥ 2.0, and the significantly DEGs were required to simultaneously meet with the absolute value of foldchange ≥ 2.0 and *p*_value ≤ 0.001. The threshold of *q*_value of ≤ 0.05 was for significantly enriched analysis of GO (Gene Ontology) and KEGG pathway.

### qRT-PCR

It validated the data of RNA-Seq sequencing by carrying out quantitative real-time PCR (qRT-PCR) on a QuantStudio 3 Real-Time PCR System. Additional file 1: Table S1 lists the oligonucleotide primers synthesized by GenerayBiotech Co., Ltd (Shanghai, China). The first strand of cDNA was synthesized using the cDNA synthesis kit (Torobo Co., Osaka, Japan). PCR amplification program was as follows: 95 °C for 1 min, and then 40 cycles at 95 °C for 15 s and 54 °C for 15 s, and 72 °C for 45 s with a SYBR Green Realtime PCR Master Mix (Torobo Co., Osaka, Japan). *ACT* (actin) was used as the internal control [29].

### Microscopic assays

The effect of taurine on mycelia morphology and structure for *M. purpureus* was assayed using scanning electron microscope (SEM) (Hitachi SU3800, Japan) and transmission electron microscope (TEM) (JEM1400, Tokyo, Japan). With the microscopic samples prepared according to the previous method [30], electron microscopic assays were carried out by Hangzhou Yanqu Information Technology Co., Ltd., China. Image-pro Plus software was used to carry out statistical analysis of SEM and TEM images.

Here, it further assayed the effect of taurine on cell membrane permeability for *M. purpureus*. Harvested at 12,000 rpm for 5 min and washed with phosphate buffer (PBS), the mycelia were stained using 10 μg/mL PI in the dark according to the method with slight modification [31]. The stained mycelia were observed using Zeiss LSM710 laser confocal microscope after washed three times with PBS. All assays were carried out in triplicate.

### Determination of MonAzPs

Extracted with a 70% (v/v) ethanol and fermentation broth (1:1) at 60 °C for 1 h, MonAzPs was determined according to the previous method with slight modification [32]. In detail, optical density (OD) of MonAzPs was separately measured at 505 nm for red, 410 nm for yellow and 470 nm for orange using L6S UV–Vis spectrophotometer (INESA Scientific Instrument Co., Ltd, Shanghai, China) after filtered with 0.22 μm filters. OD units/L was used to indicate the content of MonAzPs.

### HPLC analysis

Glucose, xylose, ethanol, taurine, furanic aldehydes and phenolic aldehydes were determined following the previous methods [5].

## Results

### Co-production of bioethanol and MonAzPs from *M. purpureus*

Genetic evolutionary analysis is illustrated in Additional file 1: Fig. S1. As the sequenced fragments of 557 bp ITS1 (OR681412) and 563 bp ITS4 (OR681413) were separately blasted in NCBI, the isolate in this study was separately shared 98.92% identity with *M. purpureus* C1, 99.91% identity with *M. purpureus* KUPM5 and 98.74% identity with *M. purpureus* ZH2. It indicated that the isolate belonged to *Monascus* genus. As well documented, *M. purpureus* strains could be used to make alcoholic beverages and produce MonAzPs [33]. Therefore, it further assessed co-production ability of bioethanol and MonAzPs for the isolate.

**Fig. 1:**
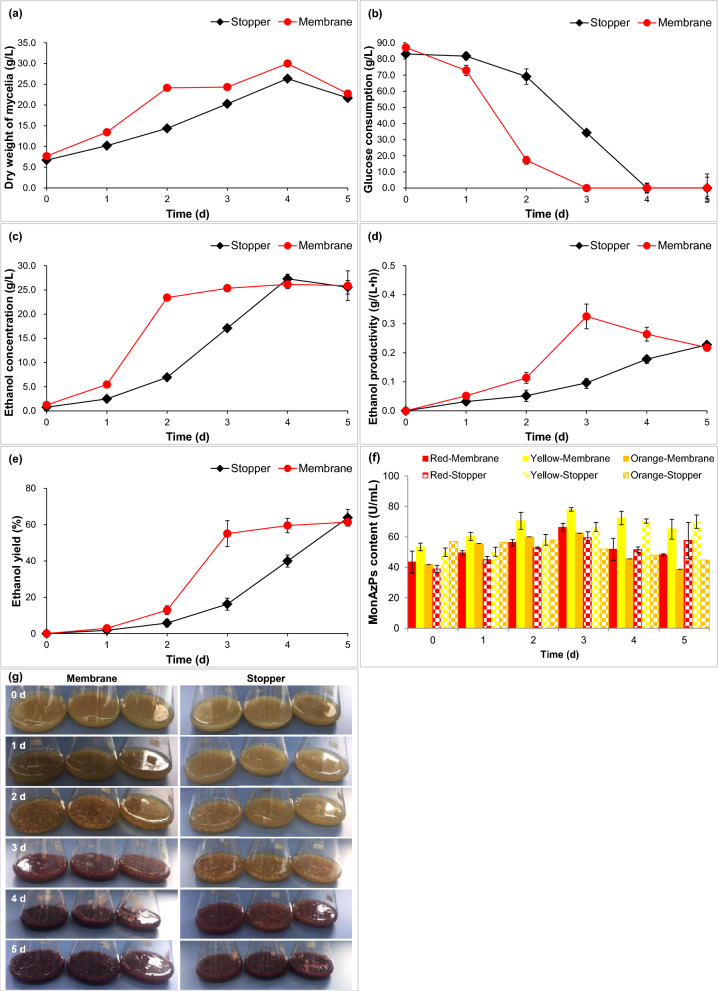
Bioethanol and MonAzPs production separately with ventilated membrane and rubber stopper for *M. purpureus*. **a** Dry weight of mycelia; **b** glucose consumption; **c** ethanol concentration; **d** ethanol productivity; **e** ethanol yield; **f** MonAzPs content; **g** flask fermentation

Here, it carried out co-production assays of bioethanol and MonAzPs for *M. purpureus* under aerobic and facultative anaerobic conditions (Fig. 1). It illustrated that mycelia growth with ventilated membrane was 1.31, 1.68, 1.20, 1.14 and 1.05 times for 1, 2, 3, 4 and 5 d than that with rubber stopper (Fig. 1a). Glucose consumption with ventilated membrane was 10.90, 4.99 and 1.78 times for 1, 2, and 3 d than that with rubber stopper (Fig. 1b). Ethanol concentration, ethanol productivity and ethanol yield for 1 d were separately augmented by 1.62, 1.62 and 1.63 times than those of the control (Fig. 1c–e). The content of red, yellow and orange MonAzPs with ventilated membrane were 66.12, 78.01 and 62.37 U/mL for 3 d at maximum content, and those with rubber stopper were 59.48, 66.51 and 52.03 U/mL, respectively (Fig. 1f, g). Herein, it achieved better co-production ability of bioethanol and MonAzPs under aerobic conditions than that under facultative anaerobic conditions for *M. purpureus*.

### Taurine promoted co-production of bioethanol and MonAzPs from *M. purpureus*

**Fig. 2:**
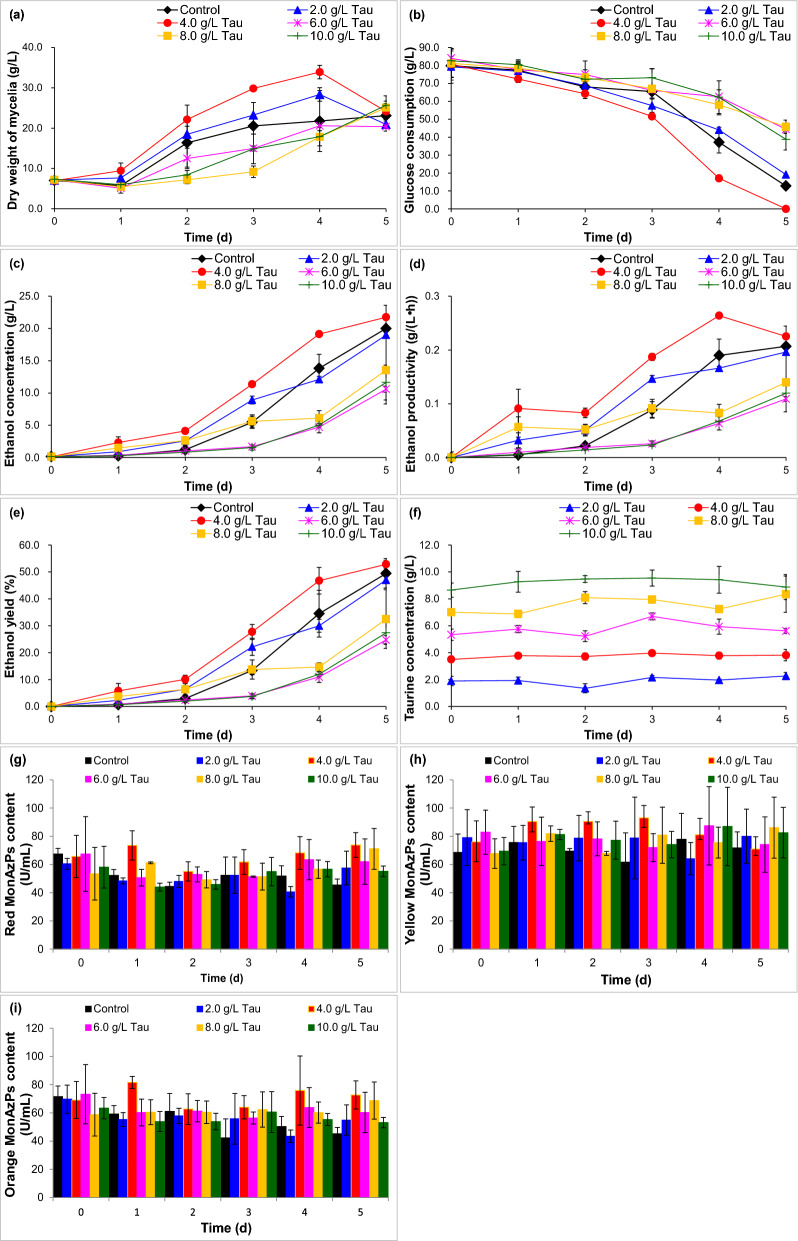
The effect of taurine (Tau) on bioethanol and MonAzPs fermentability in synthetic medium for *M. purpureus*. **a** Dry weight of mycelia; **b** glucose consumption; **c** ethanol concentration; **d** ethanol productivity; **e** ethanol yield;** f** taurine concentration; **g** red MonAzPs content; **h** yellow MonAzPs content; **i** orange MonAzPs content

Here, it assayed the effect of taurine on co-production ability of bioethanol and MonAzPs in SM with ventilated membrane for *M. purpureus* (Fig. 2). Mycelia growth for 2.0 g/L taurine was 1.34, 1.13, 1.13 and 1.30 times for 1, 2, 3 and 4 d than that of the control (Fig. 2a), and mycelia growth for 4.0 g/L taurine was 1.65, 1.35, 1.45 and 1.56 times than that of the control. No obvious promotion of mycelia growth was determined for 6.0 g/L, 8.0 g/L and 10.0 g/L taurine. Glucose consumption for 4.0 g/L taurine was 2.31, 0.39 and 1.02 times for 1, 2, and 3 d than that of the control (Fig. 2b), however, glucose consumption of 2.0 g/L taurine was 1.50 times just for 3 d. Ethanol concentration, ethanol productivity and ethanol yield for 4.0 g/L taurine were separately increased by 8.59, 9.00 and 8.76 times for 1 d than that of the control (Fig. 2c–e). *M. purpureus* consumed extremely small quantities of taurine (Fig. 2f). Compared with the control (52.22, 75.54 and 55.28 U/L), just 4.0 g/L taurine contributed to an increase by 40.71% for red MonAzPs, 19.68% for yellow MonAzPs and 47.63% for orange MonAzPs for 1 d at maximum content (Fig. 2g–i). Herein, it certainly confirmed that 4.0 g/L taurine reinforced co-production of bioethanol and MonAzPs for *M. purpureus*.

**Fig. 3:**
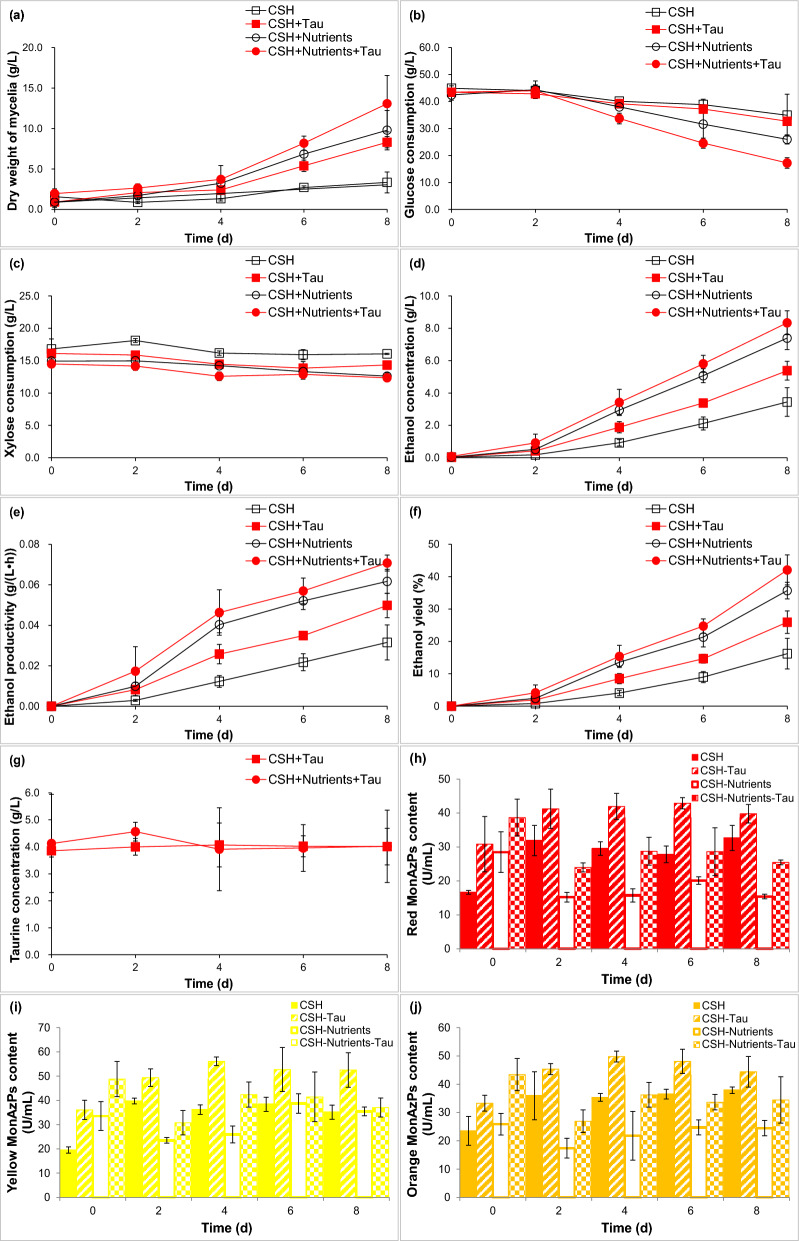
The effect of taurine on bioethanol and MonAzPs fermentability in CSH for *M. purpureus*. **a** Dry weight of mycelia; **b** glucose consumption; **c** xylose consumption; **d** ethanol concentration; **e** ethanol productivity; **f** ethanol yield; **g** taurine concentration; **h** red MonAzPs content; **i** yellow MonAzPs content; **j** orange MonAzPs content

It further investigated the effect of 4.0 g/L taurine on co-production of bioethanol and MonAzPs from CSH for *M. purpureus* (Fig. 3). Mycelia growth with taurine was 1.32, 2.79, 4.07 and 3.07 times for 2, 4, 6 and 8 d than that of the control, and mycelia growth with nutrients and taurine was 2.94, 2.16, 2.53 and 1.91 times than that of the control (Fig. 3a). Glucose consumption with taurine was 1.03 and 1.08 times for 6 and 8 d than that of the control, and glucose consumption with nutrients and taurine was 2.21, 1.75 and 1.59 times for 4, 6 and 8 d than that of the control (Fig. 3b). Xylose consumption with taurine was 2.64, 2.59 and 2.38 times for 4, 6 and 8 d than that of the control, and xylose consumption with nutrients and taurine was 2.65 times for 4 d than that of the control (Fig. 3c). Ethanol concentration, ethanol productivity and ethanol yield with taurine at the maximum were separately enhanced by 1.56, 1.58 and 1.60 times than those of the control (Fig. 3d–f). *M. purpureus* consumed extremely small quantities of taurine in CSH (Fig. 3g). Compared with the control (27.81, 36.17 and 35.33 U/L), 4.0 g/L taurine contributed to an increase of 54.19% for 6 d, 55.07% for 4 d and 40.87% for 4 d at maximum content separately for red, yellow and orange MonAzPs in CSH (Fig. 3h–j). Taurine also contributed to another increase by 82.82% for red MonAzPs, 63.25% for yellow MonAzPs and 71.15% for orange MonAzPs for 4 d in CSH with nutrients when compared with the control (15.72, 25.96 and 21.77 U/L). Therefore, it illustrated that taurine promoted co-production of bioethanol and MonAzPs from CSH for *M. purpureus*.

### Is the promotional effect of taurine universal for biorefinery fermenting strains?

It also assayed the universality of the promotional effect of taurine for the other biorefinery strains. Compared with the control, 2.0, 4.0, 6.0 and 8.0 g/L taurine obviously inhibited cell growth, glucose consumption and bioethanol production for *Z. mobilis* ZM4 (Additional file 1: Fig. S2). 1.4, 2.8, 4.2 and 5.6 g/L taurine also suppressed mycelia growth, glucose consumption, xylose consumption and itaconic acid accumulation for *A. terreus* (Additional file 1: Fig. S3). Surely, almost no taurine was consumed by the two biorefinery fermenting strains. Herein, the promotional effect of taurine was not universal for all fermenting strains.

### Transcriptional profiling of *M. purpureus* treated with taurine

Here, it further carried out de novo RNA-Seq to uncover gene transcriptional change of *M. purpureus* treated with taurine (Additional file 1: Fig. S4).

To validate the reliability of RNA-Seq sequencing data, it randomly selected 11 genes of central carbon metabolism to carry out qRT-PCR assays, such as *ACO* (aconitase), *ADH* (alcohol dehydrogenase), *CS* (citrate synthase), *ENO* (enolase), *GLK* (6-phosphofructokinase), *GND* (6-phosphogluconate dehydrogenase), *PEPCK* (phosphoenolpyruvate carboxylase), *PDH* (pyruvate dehydrogenase), *PFK* (phosphofructokinase), *PGM* (phosphoglycerate mutase) and *SCS* (succinate-CoA ligase). As shown in Additional file 1: Fig. S4a, RNA-Seq data were approximately in accordance with qRT-PCR data with 0.81 of R square ranging from − 0.48 to + 0.93, and thus indicating that the sequencing data were reliable and could be used to the further study [34].

Firstly, it illustrated that the isolate shared 83.27% similarity of *M. purpureus* in Non-Redundant (NR) Protein Sequence Database (Additional file 1: Fig. S4b). Therefore, the isolate was further identified as *M. purpureus* strain.

1148 DEGs (597 up- and 551 down-regulated genes) and 169 significantly DEGs (124 up- and 45 down-regulated genes) were screened (Additional file 1: Fig. S4c). For significant DEGs, it specially included several specific DEGs involving with major facilitator superfamily (MFS), fungal specific transcription factor domain (FSTFD), AMP-binding enzyme (ABE) and serine hydrolase (FSH1) (Additional file 1: Fig. S4c).

For GO analysis, molecular function was the most enriched (Additional file 1: Fig. S4d), followed by biological process and cellular component, and thus indicating that molecular function especially for kinase activity (GO:0016301) and translation elongation factor activity (GO:0003746) would be responsible for the promotional effect of taurine on co-production of bioethanol and MonAzPs for *M. purpureus*. For KEGG analysis, it enriched pyrimidine metabolism, purine metabolism, beta-alanine metabolism and glutathione metabolism (Additional file 1: Fig. S4e), and thus suggesting that the above pathways would relate with the promotional effect of taurine for *M. purpureus*.

Additional file 1: Fig. S4 presents the enriched metabolic pathways of taurine in KEEG database, and pyrimidine metabolism was the most enriched pathway. For *M. purpureus*, taurine was possibly catalyzed to 5-glutamyl-taurine by gamma-glutamyltranspeptidase (EC 2.3.2.2) according to gene encoding information. However, the three *GGT* genes, including *TRINITY_DN619_c0_g1_i1-C1A*, *TRINITY_DN619_c0_g1_i2-C1A* and *TRINITY_DN619_c0_g1_i3-C1A* were separately downregulated by 0.11-, 0.05- and 0.14-fold for *M. purpureus* when treated with taurine, and the other one (*TRINITY_DN9906_c0_g1_i1-S1A*) was just upregulated by 0.15-fold (Additional file 1: Fig. S5). The relative very low gene transcription of *GGT* could be used to elucidate that *M. purpureus* consumed extremely small quantities of taurine.

In conclusion, taurine contributed to gene transcription change for *M. purpureus*.

### Gene transcription of central carbon metabolism for *M. purpureus* treated with taurine

**Fig. 4:**
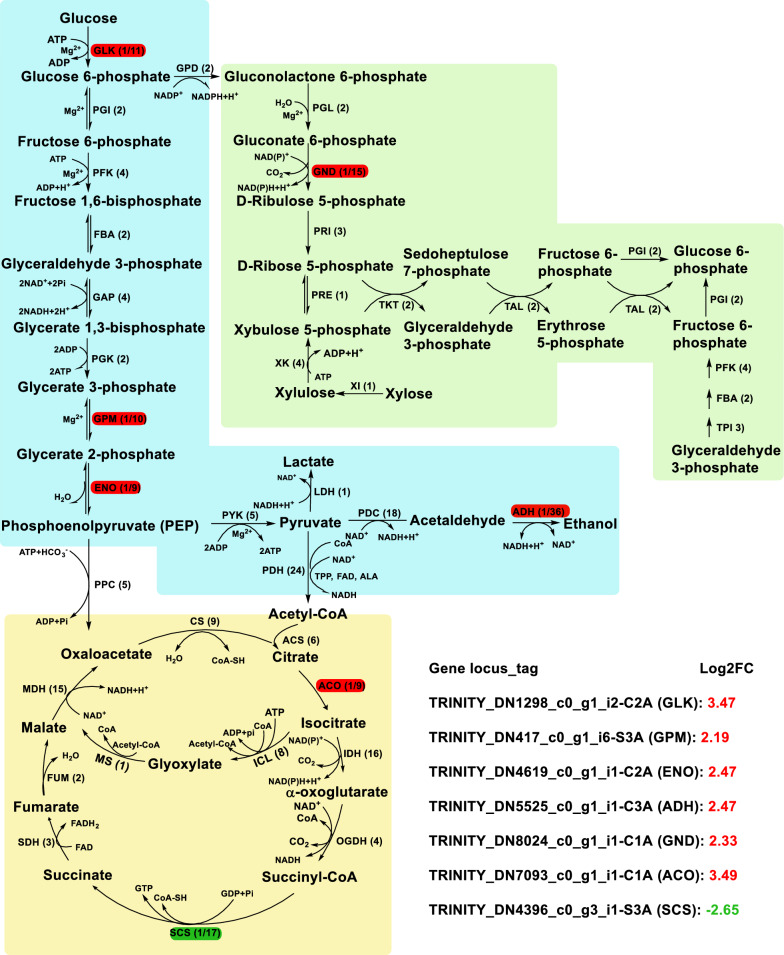
The effect of taurine on gene transcriptional profiling of central carbon metabolism for *M. purpureus*. The abbreviated enzymes were the following: ACO (aconitase), acetyl-CoA synthetase (ACS), ADH (alcohol dehydrogenase), CS (citrate synthase), ENO (enolase), FBA (fructose 1,6-bisphosphate aldolase), FUM (fumarase), GAP (glyceraldehyde 3-phosphate dehydrogenase), GLK (glucokinase), GND (6-phosphogluconate dehydrogenase), GPD (glucose-6-phosphate dehydrogenase), GPM (phosphoglycerate mutase), ICL (isocitrate lyase), IDH (isocitrate dehydrogenase), LDH (lactate dehydrogenase), MDH (malate dehydrogenase), MS (malate synthase), OGDH (α-oxoglutarate dehydrogenase), PDC (pyruvate decarboxylase), PDH (pyruvate dehydrogenase complex), PFK (phosphofructokinase), PGI (phosphoglucose isomerase), PGK (phosphoglycerate kinase), PGL (6-phosphogluconolactonase), PPC (phosphoenolpyruvate carboxylase), PRE (5-phosphate ribulose epimerase), PRI (5-phosphoribose isomerase), PYK (pyruvate kinase), SCS (succinyl-CoA synthetase), SDH (succinate dehydrogenase), TAL (transaldolase), TKT (transketolase), TPI (triose-phosphate isomerase), XI (xylose isomerase) and XK (xylulose kinase). FC indicated foldchange

Here, for the enhanced sugar consumption and ethanol accumulation for *M. purpureus* treated with taurine, gene transcription of central carbon metabolism was also investigated (Additional file 1: Table S2). Four genes, including *GLK* (glucokinase), *GPM* (phosphoglycerate mutase), *ENO* (enolase) and *ADH* (alcohol dehydrogenase) of glycolysis, were separately upregulated by 3.47-, 2.19-, 2.47- and 2.47-fold (Fig. 4), and thus indicating glycolysis pathway was partially activated by 4.0 g/L taurine. The enhanced gene expression of glycolysis could be used to partially elucidate taurine contributing to the increase of glucose consumption and ethanol accumulation for *M. purpureus*. For TCA (tricarboxylic acid cycle) pathway, just both *ACO* (aconitase) and *SCS* (succinyl-CoA synthetase) were differentially upregulated by 3.49-fold and differentially downregulated by 2.65-fold, respectively. Therefore, taurine changed gene transcriptional level of central carbon metabolism for *M. purpureus*.

### Gene transcription of MonAzPs synthesis pathway for *M. purpureus* treated with taurine

**Fig. 5:**
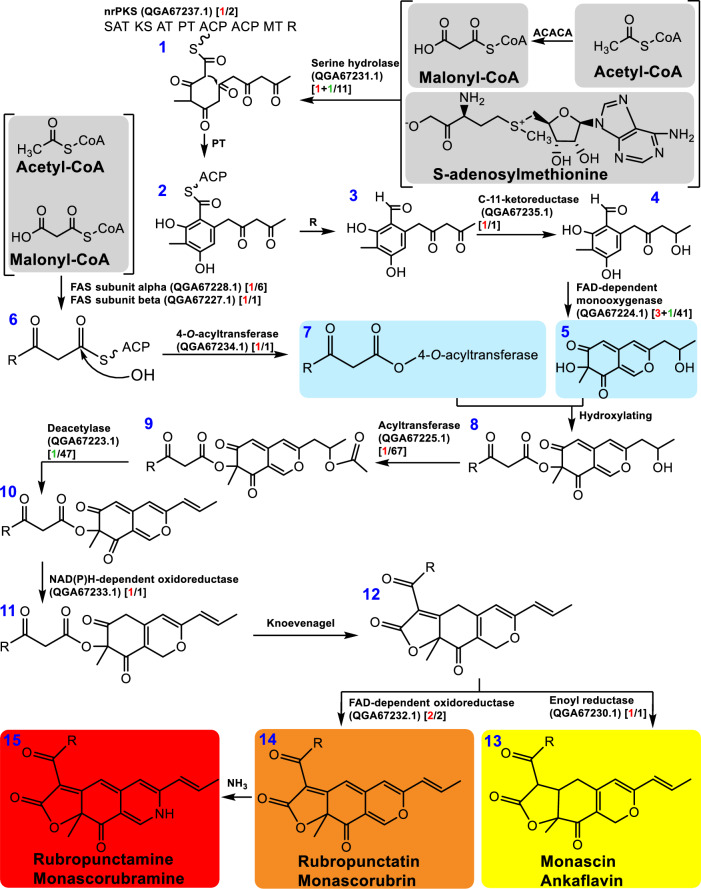
The effect of taurine on gene transcriptional profiling of MonAzPs biosynthesis for *M. purpureus*. The abbreviated enzymes were the following: ACP (acyl carrier protein), AT (acyl transferase), FAS (fatty acid synthase), KS (ketoacyl synthase), MT (methyltransferase), nrPKS (non-reducing polyketide synthase), PT (product template), release (R) and SAT (starter acyltransferase). The numbers marked red and green on the left of slash were separately the upregulated and downregulated genes in square brackets, and the ones on the right of slash were the total numbers of encoding genes. The colored red, orange and yellow rectangular indicated the three kinds of pigments, respectively. The blue Arabic figures indicated the order of the compounds

According to the previously assumed framework [11], it investigated gene transcription of MonAzPs biosynthesis pathway for *M. purpureus* (Additional file 1: Table S3; Fig. 5). The gene cluster for MonAzPs biosynthetic of the isolate was shared the nearest similarity with that of *M. purpureus* CGMCC 3.19586 (MK764694.1) after aligning the data from de novo RNA-Seq.

#### The enhanced enzyme-free intermediate biosynthesis

Three substrates malonyl-CoA, acetyl-CoA and SAM were catalyzed by serine hydrolase to synthesize hexaketide intermediate 1. One serine hydrolase gene (*TRINITY_DN2481_c0_g1_i2-C2A*) was differentially upregulated by 4.09-fold, and the other (*TRINITY_DN4092_c0_g2_i1-S2A*) was downregulated by 2.44-fold. For azaphilone polyketide biosynthesis, as MrPigG-type putative serine hydrolases, one of the non-reducing polyketide synthase (nrPKS) genes (*TRINITY_DN2093_c0_g1_i1-C2A*) responsible for the assembly of a hexaketide intermediate 1 was differentially upregulated by 4.16-fold. The first aromatic ring of the intermediate 2 happened after an aldol cyclization mediated by a product template (PT) and the resulting intermediate 3 was from the specific NADPH-dependent reductive release domain of nrPKS. C-11-ketoreductase could reduce ω-1 carbonyl to alcohol to avoid spontaneous aldol cyclization of the substrate 3 and produce the first stable enzyme-free MonAzPs intermediate 4 [33], MrPigC (*TRINITY_DN2796_c1_g1_i2-C2A*) encoding C-11-ketoreductase was just significantly differentially upregulated by 23.30-fold. In all, the above DEGs of enzyme-free intermediate biosynthesis could be used to support the promotional effect of taurine on MonAzPs production for *M. purpureus*.

#### The enhanced formation of the acylated pyran ring system

For *M. purpureus*, there were three genes encoding MrPigN (FAD-dependent monooxygenase) responsible for the hydroxylation of C-4 of the intermediate 4 and the production of pyran ring. Two genes (*TRINITY_DN3165_c0_g1_i1-S2A* and *TRINITY_DN7576_c0_g1_i1-C2A*) were upregulated by 2.07- and 4.43-fold, respectively, and one (*TRINITY_DN10522_c0_g1_i1-S1A*) was downregulated by 2.40-fold. Although *FAS* (fatty acid synthase) was one key gene for gene cluster of aflatoxin biosynthesis, the MrPigJ (FAS subunit alpha) and MrPigK (FAS subunit beta) also participated in the production of β-keto fatty acid and the side chain fatty acyl moiety of MonAzPs. For MonAzPs production, FAS could catalyze acetyl-CoA and malonyl-CoA to produce intermediate 6. Here, *TRINITY_DN1_c0_g3_i2-C2A* (FAS subunit alpha) and *TRINITY_DN1250_c0_g1_i1-C2A* (FAS subunit beta) were differentially upregulated by 4.20- and 4.16-fold, respectively. The gene (*TRINITY_DN7828_c0_g1_i1-C2A*) encoding MrPigD 4-*O*-acyltransferase catalyzing intermediate 6 to produce intermediate 7 was upregulated by 3.99-fold. The two intermediates 5 and 7 were hydroxylated and synthesized intermediate 8. Acyltransferase and deacetylase, predicted the orthologous protein relatives widespread for ascomycete fungi and synthesized the putative *O*-11 acetyl intermediate, were related with the elimination of the ω-1 alcohol. *TRINITY_DN1218_c0_g1_i4-C1A* encoding MrPigM catalyzing intermediate 8 to produce intermediate 9 and *TRINITY_DN10365_c0_g1_i1-S1A* encoding MrPigO catalyzing intermediate 9 to produce intermediate 10 were upregulated by 3.25-fold and downregulated by 3.64-fold, respectively. *TRINITY_DN9652_c0_g2_i1-C2A* was significantly differentially upregulated by 4.85-fold, which encodes NAD(P)H-dependent oxidoreductase (MPsGeE) catalyzing intermediate 10 to produce the resulting intermediate 11. Herein, the augmented gene expression of acylated pyran ring system could be used to elucidate the promotional effect of taurine on MonAzPs production for *M. purpureus*.

#### The enhanced MonAzPs synthesis

Knoevenagel cyclization of intermediate 12 led to the yellow, orange and red MonAzPs. Enoyl reductase (MPsGeH) catalyzed intermediate 12 to produce yellow pigments 13, and its encoding gene (*TRINITY_DN4418_c0_g1_i1-C2A*) was upregulated by 3.42-fold. Orange pigments 14 could be directly synthesized using intermediate 12 with FAD-dependent oxidoreductase, and the two encoding genes *TRINITY_DN9652_c0_g1_i1-C2A* and *TRINITY_DN7801_c0_g1_i1-C2A* were separately upregulated by 3.76- and 5.01-fold. The classical red pigments 15 originated from orange pigments 14 using endogenous amines of media (especially amino acids). Totally, the subsequent stage for MonAzPs synthesis was also enhanced at gene transcriptional level.

#### The enhanced transport and regulation of MonAzPs biosynthesis

Transport and regulation of MonAzPs biosynthesis were involved with transcription factor (MPsGeB and MPsGeI), ankyrin repeat protein (MPsGeL) and MFS multidrug transporter (MPsGeP). Here, it illustrated that *TRINITY_DN9874_c0_g1_i1-C2A* (MPsGeB) was upregulated by 2.17-fold. Among 302 MPsGeI encoding genes, seven transcription factor genes including *TRINITY_DN1236_c0_g1_i14-C1A*, *TRINITY_DN2716_c0_g1_i12-C2A*, *TRINITY_DN272_c0_g1_i13-S1A*, *TRINITY_DN272_c0_g1_i20-S1A*, *TRINITY_DN272_c0_g1_i9-S1A*, *TRINITY_DN35_c0_g1_i7-C2A* and *TRINITY_DN5238_c0_g1_i1-C2A*, were upregulated by 7.14, 2.37, 20.85, 21.50, 4.69, 20.99 and 2.25-fold, respectively, and another six genes including *TRINITY_DN2244_c0_g1_i2-C3A*, *TRINITY_DN2359_c0_g1_i5-S3A*, *TRINITY_DN2468_c0_g1_i1-C2A*, *TRINITY_DN3550_c0_g1_i1-S3A*, *TRINITY_DN3869_c0_g4_i1-S1A* and *TRINITY_DN4438_c0_g2_i1-S3A* were separately downregulated by 2.07, 7.23, 6.41, 2.16, 4.61 and 5.91-fold. No DEGs were found for MPsGeL. Two genes *TRINITY_DN2118_c0_g1_i1-C2A* and *TRINITY_DN2118_c0_g1_i2-C2A* (MPsGeP), a hypothetical protein in NR database annotation and MFS multidrug transporter in pfam database annotation, were upregulated by 5.53- and 4.30-fold, respectively. In all, the increased gene expression on transport and regulation could also be used to support the promotional effect of taurine on MonAzPs production for *M. purpureus*.

In conclusion, taurine contributed to the increase of gene transcription for MonAzPs biosynthesis to some extent for *M. purpureus*, especially with the upregulated genes dominant.

### Microscopic assays of *M. purpureus* treated with taurine

**Fig. 6:**
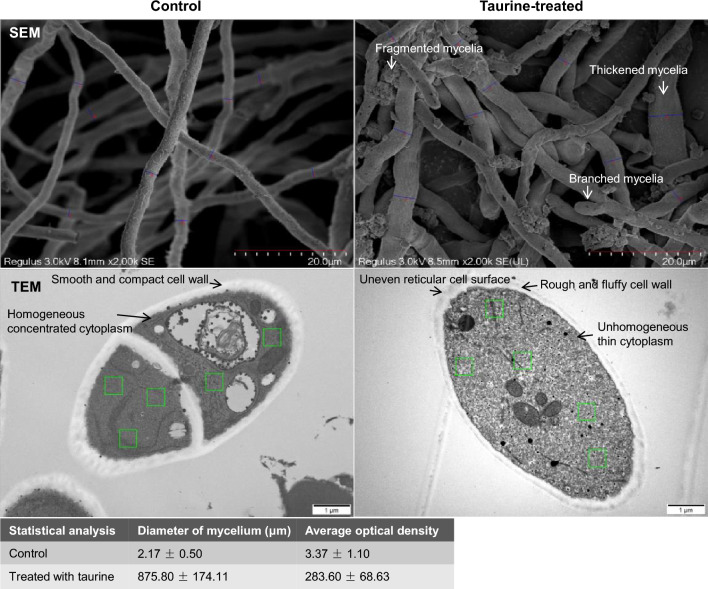
SEM and TEM images of *M. purpureus* treated with taurine. It used Image-pro Plus software to analyze the dimeter of mycelium for SEM images (*n* = 10) and the average optical density of mycelium for TEM images (*n* = 5)

**Fig. 7:**
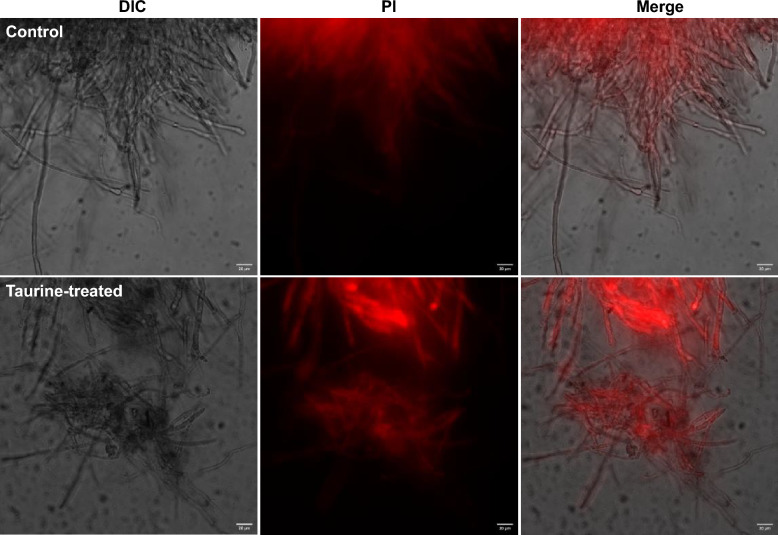
Cell membrane permeability of mycelium for *M. purpureus* treated with taurine. Differential interference contrast (DIC) and propidium iodide (PI) were separately for bright field channel and UV channel, and merge indicated both bright light channel and UV channel. Scale bars indicated 20 μm

Here, it also investigated mycelia morphology and structure for *M. purpureus* treated with taurine (Fig. 6). As Fig. 6 shows, the average diameter was separately 2.17 and 3.37 μm for the control and the taurine-treated mycelia. Compared with the control, the mycelia treated with taurine were thickened and branched. Most importantly, the mycelia treated with taurine were also fragmented according to SEM images. According to the electronic cloud density of TEM images, cell wall of mycelia for the control was smooth and compact, however, the rough fluffy cell wall of mycelia treated with taurine was of uneven cell surface with a reticular structure. Compared with mycelia of the control filled with homogeneous and concentrated cytoplasm, cell membrane of the taurine-treated mycelia was filled with unhomogeneous and thin cytoplasm, and thus was supported by statistical analysis that the average optical density was separately 875.80 and 283.60 for the control and the taurine-treated mycelia (Fig. 6). Fluorescent dye penetration assays showed that propidium iodide (PI) occurred at the sides of cell membrane for *M. purpureus* treated with taurine (Fig. 7), and thus indicated that taurine changed cell membrane structure and increased cell membrane permeability of mycelia to some extent. Herein, it illustrated that taurine increased cell membrane permeability by changing the structure of cell membrane for *M. purpureus*.

## Discussion

*M. purpureus* was known for MonAzPs production and wine starters of alcoholic beverages [33, 35, 36]. Here, it was for the first time the effect of taurine on co-production of bioethanol and MonAzPs for *M. purpureus* was investigated.

### Fermentation assays for co-production of bioethanol and MonAzPs for *M. purpureus*

Here, more bioethanol and MonAzPs were obtained in SM under aerobic conditions, and thus was in accordance with that *M. purpureus* strains usually fermented the substrates under aerobic conditions [37]. Although with good tolerance to ethanol [38], the co-production ability of bioethanol and MonAzPs was weakened in CSH than that in SM for *M. purpureus* in this study. It predicted that the weakening was derived from the inhibitory effect of furanic aldehydes and phenolic aldehydes in CSH, and thus was supported by the documented inhibitory effect from rice husk on *M. Purpureus* M523 [39]. We found that exogenous taurine increased co-production ability of bioethanol and MonAzPs in CSH, and thus was predicted that taurine might endow *M. purpureus* with the potential of stress tolerance against the inhibitors the same with the enhanced effect of taurine on *Trifolium alexandrinum* and pea (*Pisum sativum* L.) [40, 41]. Furthermore, the isolated *M. purpureus* could utilize xylose from CSH to co-produce bioethanol and MonAzPs (Fig. 3b, c), and thus was in accordance with the ability of filamentous fungi to utilize xylose from the hydrolysis of lignocellulosic biomass to produce ethanol and other biochemicals [42].

### Gene transcription for *M. purpureus* treated with taurine

De novo RNA-Seq assays uncovered a series of key genes for *M. purpureus* treated with taurine. (1) For DEGs, the enhanced effect of product accumulation for MFS, transcription factor and FSH1 had been proofed for *Monascus* strains [16, 43, 44]. The enriched kinase activity was supported by cyclic AMP (cAMP)-protein kinase A (PKA) signaling pathway in GO analysis for MonAzPs production of *M. purpureus* [45]. Therefore, the DEGs and the significant DEGs in response to taurine would be the potential synthetic biology tools to enhance co-production of bioethanol and MonAzPs for *M. purpureus* [42]. (2) For central carbon metabolism, the enhanced gene transcription, such as *GLK*, *GPM*, *ENO* and *ADH* of glycolysis, could be used to explain the elevated glucose consumption in Figs. 2b and 3b. Additionally, de novo RNA-Seq provided the gene transcriptional clue for xylose isomerase (XI) and xylulose kinase (XK) for xylose utilization in Fig. 3c for *M. purpureus*, and the xylose metabolism pathway was the same with oleaginous fungus *Mucor circinelloides* [46]. (3) For MonAzPs biosynthesis, among 15 steps of biocatalytic reactions shown in Fig. 5, 15 DEGs were screened, such as serine hydrolases (2), C-11-ketoreductase (1), FAD-dependent monooxygenase (4), 4-*O*-acyltransferase (1), deacetylase (1), NAD(P)H-dependent oxidoredutase (1), FAD-dependent oxidoredutase (2), enoyl reductase (1) and FAS subunit (2). The change of gene transcription for MonAzPs biosynthesis was in response to taurine for *M. purpureus*. Of course, for azaphilone polyketide biosynthesis, although the deficiency of serine hydrolase encoding genes severely reduced MonAzPs formation, the role of the MrPigG-type putative serine hydrolases was controversial [44, 47]. Here, the differential expression of serine hydrolase genes was in response to taurine. (4) For taurine utilization, it was thought that the isolated *M. purpureus* consumed extremely small quantities of taurine. The low gene expression level could be used to elucidate the phenomenon of extremely small quantities of taurine consumption for *M. purpureus* (Figs. 2d and 3e). After all, taurine is a micronutrient. Here, taurine concentration was determined using HPLC and represented as gram per liter, while it found nano-gram per microliter taurine was for the serum of *Mus musculus* and *Macaca mulatta* and micromole per liter taurine was for the serum of *Homo sapiens* [48]. Therefore, methods to detect lower concentration of taurine should be used to assess its utilization.

### Morphology and structure of mycelia for *M. purpureus* treated with taurine

(1) For morphology of mycelia, exogenous taurine brought about the fragmented, thickened and branched mycelia for *M. purpureus*, and thus indicating that taurine facilitated mycelia growth which was in accordance with the increased dry weight of mycelia in Figs. 1a, 2a, 3a; (2) for structure of mycelia, the rough fluffy cell wall suggested an increase of cell surface area by modification of taurine, and thus was in accordance with the effect of nonionic surfactant on *M. anka* [49]. The increased cell surface area would provide the robust assimilation ability of nutrients (sugars) for *M. purpureus*, and thus supported the enhanced glucose and xylose consumption in fermentation assays (Figs. 1b, 2b, 3b, c). For structure of mycelia, taurine increased cell membrane permeability for *M. purpureus*, and thus indicated the change of cell membrane structure. The enhanced cell membrane permeability would facilitate the delivery of intracellular metabolites (ethanol and MonAzPs) to the extracellular broth and extracellular substrates (glucose and xylose) to the intracellular, and thus was supported by the elevated effect of nonionic surfactant on *M. anka* [50, 51]. The enhanced cell membrane permeability could also be used to elucidate the facilitated sugar consumption and ethanol and MonAzPs accumulation; and (3) for the effect of concentration of taurine on morphology and structure of mycelia, 4.0 g/L taurine was the optimal alternative for co-production of bioethanol and MonAzPs. Here, it was predicted 4.0 g/L taurine as a threshold to increase cell membrane permeability by changing cell membrane structure.

### The relationship between gene transcription and cell membrane permeability

This study also tried to establish the relationship between 169 significantly DEGs and cell membrane permeability (Additional file 1: Table S4). ABC transporter (*TRINITY_DN2401_c0_g2_i2-S3A*), efflux pumps expressed in cell membrane, upregulated by 7.25-fold for *M. purpureus* treated with taurine, was predicted tightly associated with cell membrane permeability, and thus was supported by the usefulness of ABC transporter in terms of membrane permeability [52, 53]. Interestingly, cytochrome P450 (*TRINITY_DN8651_c0_g1_i1-C2A*), upregulated by 2.09-fold for *M. purpureus*, might regulate membrane permeability, and thus corresponded with the effect on plasma membrane permeability of metal ion [54]. Major facilitator superfamily (MFS) transporter was also connected with permeability [55]. In our study, eight MFS encoding genes including *TRINITY_DN326_c0_g1_i1-C2A*, *TRINITY_DN679_c0_g1_i1-C2A*, *TRINITY_DN679_c0_g1_i2-C2A*, *TRINITY_DN679_c0_g1_i3-C2A*, *TRINITY_DN679_c0_g1_i4-C2A*, *TRINITY_DN679_c0_g1_i7-C2A*, *TRINITY_DN326_c0_g1_i5-C2A* and *TRINITY_DN326_c0_g1_i4-C2A* were upregulated 4.72, 7.96, 3.64, 3.77, 21.12, 22.02, 7.17 and 21.24-fold, respectively. The other three MFS genes such as *TRINITY_DN2036_c0_g1_i1-S2A*, *TRINITY_DN1569_c0_g1_i4-C2A* and *TRINITY_DN2036_c0_g1_i5-S2A* were separately downregulated by 4.60, 2.21 and 4.70-fold. Therefore, we speculated that MFS might be responsible for the enhanced cell membrane permeability for *M. purpureus* treated with taurine.

In conclusion, taurine reinforced co-production of bioethanol and MonAzPs for *M. purpureus*. This work would provide a taurine-based activator to optimize fermentation process for mass accumulation of value-added biofuels from lignocellulosic biomass.

## Conclusions

This work presented a novel efficient taurine-based co-production system of bioethanol and MonAzPs from CSH for *M. purpureus* that consumed extremely small quantities of taurine. Exogenous taurine contributed to the improved gene transcriptional level of glycolysis and MonAzPs biosynthesis and cell membrane permeability through changing cell membrane structure of *M. purpureus*. To our knowledge, this is the first report for taurine-based co-production of bioethanol and MonAzPs from CSH for *M. purpureus*.

### Supplementary Information

**Additional file 1:** **Table S1.** Primers for qPCR in this study. **Table S2.** The gene expression level of central carbon metabolism for *M. purpureus* treated with taurine. **Table S3.** The gene expression level of MonAzPs biosynthesis pathway for *M. purpureus* treated with taurine. **Table S4.** The 169 significant DEGs for *M. purpureus* treated with taurine. **Figure S1.** Genetic evolutionary analysis for the isolated fungus strain. **a** The colony of the isolate. **b** Bootstrap consensus tree. The numbers on the branch indicated the node statistics. **Figure S2.** The effect of taurine on bioethanol fermentability for *Z. mobilis* ZM4. **a** Cell growth; **b** Glucose consumption; **c** Ethanol concentration; **d** Taurine concentration. **Figure S3.** The effect of taurine on itaconic acid fermentability for *A. terreus*. **a** Dry weight of mycelia; **b** Glucose consumption; **c** Xylose consumption; **d** Itaconic acid concentration; **e** Taurine concentration. **Figure S4.** Transcriptional profiling for bioethanol and MonAzPs production of *M. purpureus* treated with taurine. **a** Validation of RNA-Seq data using qRT-PCR; **b** Homologous species distribution of the isolate in Non-Redundant Protein Sequence Database; **c** The DEGs; **d** The relative expression level of the most enriched significant DEGs; **e** GO analysis; **f** KEGG pathway analysis. **Figure S5.** Taurine metabolism pathway and its gene expression level.

## Data Availability

RNA-Seq sequence data were deposited in the GEO database at NCBI. (https://www.ncbi.nlm.nih.gov/geo/query/acc.cgi?acc=GSE248985) in the GEO database at NCBI.

## Abbreviations

ABE: AMP-binding enzyme
ACO: Aconitase
ADH: Alcohol dehydrogenase
CSH: Corn stover hydrolysates
DEGs: Differentially expressed genes
ENO: Enolase
FSH1: Serine hydrolase
FSTFD: Fungal specific transcription factor domain
GGT: Gamma-glutamyltranspeptidase
GLK: Glucokinase
GO: Gene Ontology
GPM: Phosphoglycerate mutase
HPLC: High performance liquid chromatography
ITS1: Internal transcribed spacer
MFS: Major facilitator superfamily
MonAzPs: Monascus azaphilone pigments
NR: Non-redundant
nrPKS: Non-reducing polyketide synthase
OD: Optical density
PDA: Potato-dextrose agar
PI: Propidium iodide
PT: Product template
qRT-PCR: Quantitative real-time PCR
SCS: Succinyl-CoA synthetase
TCA: Tricarboxylic acid cycle
RM: Rich medium
SEM: Scanning electron microscope
TEM: Transmission electron microscope
XI: Xylose isomerase
XK: Xylulose kinase
