# Supplementary material for: Taurine-mediated gene transcription and cell membrane permeability reinforced co-production of bioethanol and Monascus azaphilone pigments for a newly isolated Monascus purpureus
Source: Biotechnol Biofuels Bioprod. 2024 May 3;17:59. doi: 10.1186/s13068-024-02511-7 (PMC11069175; doi:10.1186/s13068-024-02511-7)
Supplement: Supplementary file 1 — Additional file 1: Table S1. Primers for qPCR in this study. Table S2. The gene expression level of central carbon metabolism for M. purpureus treated with taurine. Table S3. The gene expression level of MonAzPs biosynthesis pathway for M. purpureus treated with taurine. Table S4. The 169 significant DEGs for M. purpureus treated with taurine. Figure S1. Genetic evolutionary analysis for the isolated fungus strain. a The colony of the isolate. b Bootstrap consensus tree. The numbers on the branch indicated the node statistics. Figure S2. The effect of taurine on bioethanol fermentability for Z. mobilis ZM4. a Cell growth; b Glucose consumption; c Ethanol concentration; d Taurine concentration. Figure S3. The effect of taurine on itaconic acid fermentability for A. terreus. a Dry weight of mycelia; b Glucose consumption; c Xylose consumption; d Itaconic acid concentration; e Taurine concentration. Figure S4. Transcriptional profiling for bioethanol and MonAzPs production of M. purpureus treated with taurine. a Validation of RNA-Seq data using qRT-PCR; b Homologous species distribution of the isolate in Non-Redundant Protein Sequence Database; c The DEGs; d The relative expression level of the most enriched significant DEGs; e GO analysis; f KEGG pathway analysis. Figure S5. Taurine metabolism pathway and its gene expression level. [file 13068_2024_2511_MOESM1_ESM.docx]

**Additional file**

**Table S1** Primers for qPCR in this study.

| Gene | Product | Forward primer (5'-3') | Reverse primer (5'-3') |
| --- | --- | --- | --- |
| *Actin* | Actin | TTGCGTCCACGGAGTTTT | CCGCTCGATCTCCACGAG |
| *ACO* | Aconitase | ATCAATAAGCCCCGGCCT | CGATCCCGGTCCCGTATA |
| *ADH* | Alcohol dehydrogenase | CGAGATGGATTCGGCAAC | AGGTCGCCAACGTTGATG |
| *CS* | Citrate synthase | CCAGGTTCCGGCCTTTCT | TTTCACCCGCGGATCAAC |
| *ENO* | Enolase | TGCACCGGTTGGAGCAAC | AATTCCACCAGCGGCAGA |
| *GLK* | 6-Phosphofructokinase | GGCCAGACGTCCTCGAAG | GTCCTTCGTCCAGCGCTG |
| *GND* | 6-Phosphogluconate dehydrogenase | TACTGCTGCTTCCTGGCC | AGTTGCCCAGGTGGATGT |
| *PDH* | Pyruvate dehydrogenase | GGGAAAGCTCGCTGGACA | AGTTCAAGACTGCCGGCC |
| *PEPCK* | Phosphoenolpyruvate carboxylase | GCTTGCTAAGGCCGAGTA | TCCAGCTGTGTCTGGGGT |
| *PFK* | Phosphofructokinase | ATGATCCGGGAGGAAGCA | CACGGTCCATTGGCGATG |
| *PGM* | Phosphoglycerate mutase | TCACCTTCAGAGCGTCGG | CTCCTTCAGGACAGCCGT |
| *SCS* | Succinate-CoA ligase | ATGCCGCGTTCAGGAGTT | CAATCCCGTCCTTTTCGG |

**Table S2** The gene expression level of central carbon metabolism for *M. purpureus* treated with taurine.

| Product | Gene | Gene locus_tag | Log_2_Foldchange | *p*_value |
| --- | --- | --- | --- | --- |
| Aconitase | ACO | TRINITY_DN3024_c0_g1_i1-C3A | 0.61 | 0.13 |
|  |  | TRINITY_DN3024_c0_g1_i2-C3A | 0.70 | 0.59 |
|  |  | TRINITY_DN4453_c0_g4_i1-S2A | -0.97 | 0.08 |
|  |  | TRINITY_DN1687_c0_g3_i1-S3A | -0.08 | 0.54 |
|  |  | TRINITY_DN1708_c0_g1_i1-S3A | 0.07 | 0.76 |
|  |  | TRINITY_DN2029_c0_g1_i1-C1A | -0.05 | 0.72 |
|  |  | TRINITY_DN2619_c0_g1_i1-S1A | 0.02 | 0.82 |
|  |  | **TRINITY_DN7093_c0_g1_i1-C1A** | **3.48** | **0.38** |
|  |  | TRINITY_DN9099_c0_g1_i1-C2A | -0.85 | 0.82 |
| Acyl-CoA Synthetase | ACS | TRINITY_DN2309_c0_g1_i1-S3A | -0.14 | 0.53 |
|  |  | TRINITY_DN1136_c0_g1_i1-S3A | 0.01 | 0.96 |
|  |  | TRINITY_DN855_c0_g1_i2-C3A | 0.37 | 0.23 |
|  |  | TRINITY_DN10799_c0_g1_i1-S1A | -0.11 | 0.50 |
|  |  | TRINITY_DN706_c0_g1_i1-S3A | -0.08 | 0.32 |
|  |  | TRINITY_DN1316_c1_g1_i1-C3A | -0.21 | 0.75 |
|  |  | TRINITY_DN7474_c0_g1_i1-C2A | 0.08 | 0.55 |
|  |  | TRINITY_DN8494_c0_g1_i1-C2A | 1.60 | 0.09 |
| Alcohol dehydrogenase | ADH | TRINITY_DN3613_c0_g2_i1-C2A | 0.49 | 0.36 |
|  |  | TRINITY_DN4044_c0_g1_i1-C1A | -0.10 | 0.60 |
|  |  | TRINITY_DN5140_c0_g1_i2-C3A | -0.85 | 0.82 |
|  |  | TRINITY_DN5853_c0_g1_i1-S1A | 0.74 | 0.23 |
|  |  | TRINITY_DN10119_c0_g1_i1-C2A | 0.82 | 0.13 |
|  |  | TRINITY_DN10772_c0_g1_i1-S1A | -0.29 | 0.52 |
|  |  | TRINITY_DN1471_c0_g1_i2-C1A | -0.12 | 0.87 |
|  |  | TRINITY_DN1533_c0_g3_i1-C2A | 0.47 | 0.27 |
|  |  | TRINITY_DN1533_c0_g3_i2-C2A | 0.24 | 0.38 |
|  |  | TRINITY_DN1545_c0_g1_i2-S3A | -0.22 | 0.58 |
|  |  | TRINITY_DN1545_c0_g1_i3-S3A | 0.15 | 0.61 |
|  |  | TRINITY_DN1545_c0_g1_i4-S3A | 0.12 | 0.67 |
|  |  | TRINITY_DN1572_c0_g1_i8-C1A | 0.00 | 0.99 |
|  |  | TRINITY_DN1606_c0_g1_i1-C2A | 0.33 | 0.29 |
|  |  | TRINITY_DN1841_c0_g1_i1-C3A | 0.97 | 0.25 |
|  |  | TRINITY_DN2212_c0_g1_i1-C1A | 0.31 | 0.63 |
|  |  | TRINITY_DN2212_c0_g1_i3-C1A | 0.30 | 0.58 |
|  |  | TRINITY_DN2212_c0_g1_i5-C1A | 1.07 | 0.10 |
|  |  | TRINITY_DN2295_c0_g1_i1-S1A | -0.13 | 0.77 |
|  |  | TRINITY_DN2295_c0_g1_i2-S1A | -0.29 | 0.11 |
|  |  | TRINITY_DN2309_c0_g1_i1-C3A | -0.18 | 0.73 |
|  |  | TRINITY_DN2519_c1_g1_i1-C2A | 1.20 | 0.16 |
|  |  | TRINITY_DN2874_c0_g1_i1-C2A | -0.70 | 0.14 |
|  |  | TRINITY_DN3439_c0_g1_i1-C2A | -0.50 | 0.22 |
|  |  | TRINITY_DN3450_c0_g1_i1-S1A | 0.12 | 0.69 |
|  |  | TRINITY_DN3489_c0_g1_i1-C3A | 0.60 | 0.21 |
|  |  | TRINITY_DN4283_c0_g1_i2-S3A | -0.11 | 0.77 |
|  |  | TRINITY_DN4501_c0_g1_i2-C3A | 0.62 | 0.38 |
|  |  | TRINITY_DN4620_c0_g2_i1-C2A | 0.53 | 0.56 |
|  |  | TRINITY_DN4767_c0_g2_i1-S1A | -0.96 | 0.68 |
|  |  | TRINITY_DN5310_c0_g1_i1-S3A | 0.78 | 0.74 |
|  |  | **TRINITY_DN5525_c0_g1_i1-C3A** | **2.46** | **0.54** |
|  |  | TRINITY_DN7420_c0_g1_i1-C2A | -0.05 | 0.85 |
|  |  | TRINITY_DN7693_c0_g1_i1-S2A | 0.04 | 0.88 |
|  |  | TRINITY_DN7852_c0_g1_i1-S3A | -0.99 | 0.43 |
|  |  | TRINITY_DN9904_c0_g1_i1-C2A | 0.04 | 0.81 |
| Citrate synthase | CS | TRINITY_DN2393_c0_g1_i1-C2A | -0.01 | 0.95 |
|  |  | TRINITY_DN3919_c0_g2_i1-C2A | 1.37 | 0.05 |
|  |  | TRINITY_DN465_c0_g1_i1-S3A | 0.10 | 0.12 |
|  |  | TRINITY_DN465_c0_g1_i2-S3A | -0.79 | 0.18 |
|  |  | TRINITY_DN4898_c0_g3_i2-C1A | 0.20 | 0.90 |
|  |  | TRINITY_DN661_c0_g1_i1-S1A | -0.09 | 0.76 |
|  |  | TRINITY_DN661_c0_g1_i5-S1A | 0.81 | 0.00 |
|  |  | TRINITY_DN8970_c0_g1_i1-C3A | 0.00 | 1.00 |
|  |  | TRINITY_DN9972_c0_g1_i1-S2A | -1.59 | 0.52 |
| Enolase | ENO | TRINITY_DN439_c0_g1_i2-C2A | 0.38 | 0.31 |
|  |  | TRINITY_DN439_c0_g1_i3-C2A | 0.40 | 0.08 |
|  |  | **TRINITY_DN4619_c0_g1_i1-C2A** | **2.47** | **0.54** |
|  |  | TRINITY_DN4619_c0_g2_i1-C2A | 1.50 | 0.45 |
|  |  | TRINITY_DN520_c0_g1_i4-S2A | 0.41 | 0.31 |
|  |  | TRINITY_DN10750_c0_g1_i1-C2A | 0.35 | 0.20 |
|  |  | TRINITY_DN2227_c0_g1_i1-S3A | -0.01 | 0.96 |
|  |  | TRINITY_DN4604_c0_g2_i1-C1A | 0.71 | 0.27 |
|  |  | TRINITY_DN4872_c0_g1_i2-C2A | -0.38 | 0.78 |
| Fructose 1,6-bisphosphate aldolase | FBA | TRINITY_DN1421_c0_g1_i1-S1A | 0.42 | 0.18 |
| Fructose-bisphosphatase | FBP | TRINITY_DN1421_c0_g1_i1-S1A | 0.42 | 0.18 |
|  |  | TRINITY_DN6152_c0_g1_i1-C2A | 0.10 | 0.77 |
|  |  | TRINITY_DN1327_c0_g2_i1-S1A | 0.04 | 0.77 |
|  |  | TRINITY_DN674_c0_g1_i1-S3A | 0.18 | 0.54 |
|  |  | TRINITY_DN3486_c0_g1_i1-S1A | -0.09 | 0.52 |
|  |  | TRINITY_DN6152_c0_g1_i1-C2A | 0.10 | 0.77 |
| Fumarase |  | TRINITY_DN8932_c0_g1_i1-S3A | ND | ND |
|  |  | TRINITY_DN3136_c0_g2_i1-S1A | -0.07 | 0.00 |
| Glyceraldehyde 3-phosphate dehydrogenase | GAP | TRINITY_DN308_c0_g1_i1-C3A | 0.50 | 0.18 |
|  |  | TRINITY_DN308_c0_g1_i5-C3A | 0.30 | 0.50 |
|  |  | TRINITY_DN308_c0_g1_i6-C3A | 0.01 | 0.98 |
|  |  | TRINITY_DN369_c0_g1_i3-S3A | 0.16 | 0.70 |
| Hexokinase | GLK | **TRINITY_DN1298_c0_g1_i2-C2A** | **3.47** | **0.38** |
|  |  | TRINITY_DN1905_c0_g1_i2-S2A | 0.14 | 0.84 |
|  |  | TRINITY_DN25_c1_g1_i2-C2A | 0.26 | 0.28 |
|  |  | TRINITY_DN25_c1_g1_i3-C2A | 0.40 | 0.35 |
|  |  | TRINITY_DN25_c1_g1_i4-C2A | 0.90 | 0.18 |
|  |  | TRINITY_DN2785_c0_g1_i1-C1A | 0.32 | 0.29 |
|  |  | TRINITY_DN3658_c0_g1_i1-C2A | -0.19 | 0.71 |
|  |  | TRINITY_DN3658_c0_g1_i2-C2A | -0.02 | 0.93 |
|  |  | TRINITY_DN3877_c0_g2_i5-S2A | -0.29 | 0.25 |
|  |  | TRINITY_DN438_c2_g2_i1-S3A | 0.08 | 0.83 |
|  |  | TRINITY_DN4535_c0_g1_i1-S3A | 0.55 | 0.35 |
|  |  | TRINITY_DN4535_c0_g1_i2-S3A | 1.85 | 0.16 |
|  |  | TRINITY_DN4535_c0_g1_i3-S3A | -1.98 | 0.42 |
|  |  | TRINITY_DN4535_c0_g1_i4-S3A | -0.92 | 0.64 |
|  |  | TRINITY_DN5067_c0_g2_i1-C3A | -0.09 | 0.36 |
|  |  | TRINITY_DN741_c2_g2_i1-C1A | -0.25 | 0.32 |
|  |  | TRINITY_DN741_c2_g2_i4-C1A | 0.32 | 0.14 |
|  |  | TRINITY_DN8770_c0_g1_i1-S1A | -0.93 | 0.77 |
| 6-Phosphogluconate dehydrogenase | GND | TRINITY_DN1163_c0_g2_i1-S2A | ND | ND |
|  |  | TRINITY_DN1163_c0_g2_i3-S2A | ND | ND |
|  |  | TRINITY_DN1163_c0_g2_i4-S2A | 0.09 | 0.82 |
|  |  | TRINITY_DN1561_c0_g1_i1-S2A | 0.05 | 0.63 |
|  |  | TRINITY_DN2307_c0_g1_i5-C2A | -0.20 | 0.46 |
|  |  | TRINITY_DN2630_c0_g1_i1-C1A | -0.05 | 0.80 |
|  |  | TRINITY_DN6424_c0_g1_i1-C1A | -0.24 | 0.34 |
|  |  | TRINITY_DN6475_c0_g1_i1-C3A | 0.66 | 0.35 |
|  |  | TRINITY_DN9027_c0_g1_i1-S3A | -1.34 | 0.46 |
|  |  | TRINITY_DN338_c0_g1_i2-S3A | -0.03 | 0.90 |
|  |  | TRINITY_DN338_c0_g1_i3-S3A | -0.14 | 0.05 |
|  |  | TRINITY_DN5649_c0_g2_i1-S1A | -0.85 | 0.40 |
|  |  | **TRINITY_DN8024_c0_g1_i1-C1A** | **2.33** | **0.28** |
|  |  | TRINITY_DN1423_c0_g2_i1-S1A | -0.10 | 0.35 |
|  |  | TRINITY_DN1268_c0_g2_i1-C3A | 0.08 | 0.77 |
| Isocitrate lyase | ICL | TRINITY_DN2028_c0_g1_i1-C2A | 0.84 | 0.11 |
|  |  | TRINITY_DN3743_c0_g1_i1-S1A | 0.21 | 0.30 |
| Isocitrate dehydrogenase (NADP^+^) | IDH | TRINITY_DN1317_c1_g1_i1-C2A | 0.05 | 0.78 |
|  |  | TRINITY_DN271_c0_g1_i1-S3A | -0.19 | 0.60 |
|  |  | TRINITY_DN271_c0_g1_i6-S3A | 0.06 | 0.73 |
|  |  | TRINITY_DN3128_c0_g2_i1-S3A | 0.04 | 0.66 |
|  |  | TRINITY_DN4484_c0_g1_i2-S2A | 0.13 | 0.47 |
|  |  | TRINITY_DN6259_c0_g1_i1-S2A | 0.03 | 0.85 |
|  |  | TRINITY_DN6456_c0_g1_i1-C2A | 0.42 | 0.44 |
|  |  | TRINITY_DN9831_c0_g1_i1-C2A | 0.07 | 0.45 |
|  |  | TRINITY_DN1317_c1_g1_i1-C2A | 0.05 | 0.78 |
|  |  | TRINITY_DN271_c0_g1_i1-S3A | -0.19 | 0.60 |
|  |  | TRINITY_DN271_c0_g1_i6-S3A | 0.06 | 0.73 |
|  |  | TRINITY_DN3128_c0_g2_i1-S3A | 0.04 | 0.66 |
|  |  | TRINITY_DN4484_c0_g1_i2-S2A | 0.13 | 0.47 |
|  |  | TRINITY_DN6259_c0_g1_i1-S2A | 0.03 | 0.85 |
|  |  | TRINITY_DN6456_c0_g1_i1-C2A | 0.42 | 0.44 |
|  |  | TRINITY_DN9831_c0_g1_i1-C2A | 0.07 | 0.45 |
| Lactate dehydrogenase | LDH | TRINITY_DN8928_c0_g1_i1-S1A | 0.06 | 0.84 |
| Malate dehydrogenase (NADP^+^) | MD | TRINITY_DN1317_c1_g1_i1-C2A | 0.05 | 0.78 |
|  |  | TRINITY_DN271_c0_g1_i1-S3A | -0.19 | 0.60 |
|  |  | TRINITY_DN271_c0_g1_i6-S3A | 0.06 | 0.73 |
|  |  | TRINITY_DN3128_c0_g2_i1-S3A | 0.04 | 0.66 |
|  |  | TRINITY_DN4484_c0_g1_i2-S2A | 0.13 | 0.47 |
|  |  | TRINITY_DN6259_c0_g1_i1-S2A | 0.03 | 0.85 |
|  |  | TRINITY_DN6456_c0_g1_i1-C2A | 0.42 | 0.44 |
|  |  | TRINITY_DN9831_c0_g1_i1-C2A | 0.07 | 0.45 |
|  |  | TRINITY_DN2245_c0_g1_i1-S1A | 0.03 | 0.76 |
|  |  | TRINITY_DN359_c0_g2_i1-S1A | -0.06 | 0.68 |
|  |  | TRINITY_DN359_c0_g2_i2-S1A | -0.24 | 0.30 |
|  |  | TRINITY_DN359_c0_g2_i3-S1A | 0.00 | 0.99 |
|  |  | TRINITY_DN359_c0_g2_i4-S1A | -0.24 | 0.09 |
|  |  | TRINITY_DN6330_c0_g1_i1-S3A | 1.73 | 0.05 |
|  |  | TRINITY_DN7949_c0_g1_i1-C2A | 1.85 | 0.06 |
| Malate synthase | MS | TRINITY_DN6942_c0_g1_i1-S3A | 0.09 | 0.64 |
| α-Ketoglutarate dehydrogenase | OGDH | TRINITY_DN7557_c0_g1_i1-C2A | 0.02 | 0.80 |
|  |  | TRINITY_DN800_c0_g1_i2-S2A | 0.05 | 0.52 |
|  |  | TRINITY_DN800_c0_g1_i3-S2A | -0.17 | 0.51 |
|  |  | TRINITY_DN994_c0_g1_i1-C2A | 0.13 | 0.54 |
| Pyruvate carboxylase | PC | TRINITY_DN395_c0_g2_i1-C2A | 0.56 | 0.26 |
|  |  | TRINITY_DN395_c0_g2_i2-C2A | 0.79 | 0.23 |
|  |  | TRINITY_DN395_c0_g2_i3-C2A | 0.50 | 0.33 |
|  |  | TRINITY_DN601_c0_g1_i1-C3A | -0.24 | 0.57 |
|  |  | TRINITY_DN630_c0_g1_i3-S3A | -0.11 | 0.81 |
| Pyruvate decarboxylase | PDC | TRINITY_DN2875_c0_g1_i1-C2A | 0.17 | 0.81 |
|  |  | TRINITY_DN2875_c0_g1_i2-C2A | 0.91 | 0.33 |
|  |  | TRINITY_DN2875_c0_g1_i3-C2A | 0.31 | 0.70 |
|  |  | TRINITY_DN2875_c0_g1_i4-C2A | -0.59 | 0.17 |
|  |  | TRINITY_DN2899_c0_g2_i3-C3A | 0.34 | 0.39 |
|  |  | TRINITY_DN2964_c0_g1_i1-C2A | ND | ND |
|  |  | TRINITY_DN2964_c0_g2_i1-C2A | 1.31 | 0.14 |
|  |  | TRINITY_DN503_c0_g1_i1-C2A | -0.14 | 0.78 |
|  |  | TRINITY_DN503_c0_g1_i2-C2A | 0.48 | 0.27 |
|  |  | TRINITY_DN503_c0_g1_i3-C2A | 0.46 | 0.54 |
|  |  | TRINITY_DN503_c0_g1_i4-C2A | 0.12 | 0.78 |
|  |  | TRINITY_DN54_c0_g1_i2-C2A | -0.03 | 0.87 |
|  |  | TRINITY_DN6880_c0_g1_i1-S1A | -0.23 | 0.72 |
|  |  | TRINITY_DN779_c0_g2_i1-C2A | 0.28 | 0.52 |
|  |  | TRINITY_DN779_c0_g2_i2-C2A | 0.75 | 0.30 |
|  |  | TRINITY_DN779_c0_g2_i3-C2A | 0.87 | 0.23 |
|  |  | TRINITY_DN8800_c0_g1_i1-C3A | -0.09 | 0.47 |
|  |  | TRINITY_DN9_c0_g1_i1-S2A | -0.20 | 0.27 |
|  |  | TRINITY_DN2502_c0_g1_i1-S1A | -0.01 | 0.93 |
| Pyruvate dehydrogenase complex | PDH | TRINITY_DN353_c0_g1_i2-S2A | 0.02 | 0.88 |
|  |  | TRINITY_DN1675_c0_g1_i1-S1A | 0.02 | 0.81 |
|  |  | TRINITY_DN426_c0_g2_i6-S3A | 0.77 | 0.23 |
|  |  | TRINITY_DN426_c0_g3_i1-S3A | -0.38 | 0.85 |
|  |  | TRINITY_DN494_c0_g1_i2-S2A | -0.42 | 0.77 |
|  |  | TRINITY_DN494_c0_g1_i6-S2A | 0.67 | 0.60 |
|  |  | TRINITY_DN781_c0_g3_i3-C3A | 0.43 | 0.58 |
|  |  | TRINITY_DN875_c0_g2_i1-S1A | 0.31 | 0.58 |
|  |  | TRINITY_DN931_c0_g1_i2-C1A | -0.20 | 0.54 |
|  |  | TRINITY_DN1968_c0_g1_i1-C3A | 0.53 | 0.47 |
|  |  | TRINITY_DN2800_c0_g1_i1-C1A | -0.02 | 0.83 |
|  |  | TRINITY_DN4541_c0_g1_i1-S3A | -0.12 | 0.36 |
|  |  | TRINITY_DN325_c0_g1_i3-S1A | ND | ND |
|  |  | TRINITY_DN4434_c0_g1_i1-S1A | 0.01 | 0.91 |
|  |  | TRINITY_DN501_c0_g1_i1-S3A | ND | ND |
|  |  | TRINITY_DN501_c0_g1_i2-S3A | 0.59 | 0.08 |
|  |  | TRINITY_DN5511_c0_g1_i1-C3A | 0.55 | 0.65 |
|  |  | TRINITY_DN651_c0_g1_i3-C3A | -0.53 | 0.01 |
|  |  | TRINITY_DN651_c0_g1_i5-C3A | 0.16 | 0.58 |
|  |  | TRINITY_DN9934_c0_g1_i1-S1A | -0.05 | 0.59 |
|  |  | TRINITY_DN1968_c0_g1_i1-C3A | 0.53 | 0.47 |
|  |  | TRINITY_DN2800_c0_g1_i1-C1A | -0.02 | 0.83 |
|  |  | TRINITY_DN4541_c0_g1_i1-S3A | -0.12 | 0.36 |
| Phosphofructokinase-1 | PFK | TRINITY_DN112_c0_g1_i1-C1A | 0.60 | 0.14 |
|  |  | TRINITY_DN112_c0_g1_i3-C1A | 0.35 | 0.42 |
|  |  | TRINITY_DN236_c0_g1_i1-C2A | 1.05 | 0.17 |
|  |  | TRINITY_DN236_c0_g1_i3-C2A | 0.53 | 0.32 |
| Phosphohexose isomerase | PGI | TRINITY_DN5927_c0_g1_i1-C1A | -0.14 | 0.20 |
|  |  | TRINITY_DN8515_c0_g1_i1-C2A | 0.22 | 0.48 |
| Phosphoglycerate kinase | PGK | TRINITY_DN1525_c0_g1_i1-C1A | 0.32 | 0.30 |
|  |  | TRINITY_DN1852_c0_g1_i1-C2A | 0.66 | 0.35 |
| Lactonase/gluconolactonase | PGL | TRINITY_DN5927_c0_g1_i1-C1A | -0.14 | 0.20 |
|  |  | TRINITY_DN8515_c0_g1_i1-C2A | 0.22 | 0.48 |
| Phosphoglycerate mutase | PGM | TRINITY_DN2100_c1_g1_i1-C1A | -0.07 | 0.39 |
|  |  | TRINITY_DN417_c0_g1_i2-S3A | 0.46 | 0.10 |
|  |  | TRINITY_DN417_c0_g1_i4-S3A | -0.04 | 0.82 |
|  |  | TRINITY_DN417_c0_g1_i5-S3A | 0.65 | 0.04 |
|  |  | TRINITY_DN7001_c0_g1_i1-C2A | -0.01 | 0.88 |
|  |  | TRINITY_DN312_c0_g1_i10-C2A | 1.57 | 0.20 |
|  |  | TRINITY_DN417_c0_g1_i3-S3A | -0.02 | 0.99 |
|  |  | **TRINITY_DN417_c0_g1_i6-S3A** | **2.19** | **0.38** |
|  |  | TRINITY_DN450_c0_g1_i7-S1A | -0.19 | 0.22 |
|  |  | TRINITY_DN19_c1_g1_i1-S3A | 0.05 | 0.74 |
| Phosphoenolpyruvate carboxykinase | PPC | TRINITY_DN395_c0_g2_i1-C2A | 0.56 | 0.26 |
|  |  | TRINITY_DN395_c0_g2_i2-C2A | 0.79 | 0.23 |
|  |  | TRINITY_DN395_c0_g2_i3-C2A | 0.50 | 0.33 |
|  |  | TRINITY_DN601_c0_g1_i1-C3A | -0.24 | 0.57 |
|  |  | TRINITY_DN630_c0_g1_i3-S3A | -0.11 | 0.81 |
| Phosphopentose isomerase | PRI | TRINITY_DN430_c0_g1_i3-C3A | 0.07 | 0.97 |
|  |  | TRINITY_DN553_c0_g1_i2-S3A | -0.04 | 0.77 |
|  |  | TRINITY_DN553_c0_g1_i3-S3A | -0.19 | 0.23 |
| Ribulose-phosphate 3-epimerase | PRE | TRINITY_DN10643_c0_g1_i1-C2A | 0.02 | 0.87 |
| Pyruvate kinase | PYK | TRINITY_DN1627_c0_g1_i1-S2A | -0.06 | 0.77 |
|  |  | TRINITY_DN2023_c0_g1_i1-C2A | 0.58 | 0.10 |
|  |  | TRINITY_DN779_c0_g1_i1-C1A | -0.01 | 0.94 |
|  |  | TRINITY_DN832_c0_g1_i2-S1A | -0.07 | 0.77 |
| Succinyl-CoA synthetase | SCS | TRINITY_DN1729_c0_g1_i1-C2A | 0.97 | 0.47 |
|  |  | TRINITY_DN2216_c0_g2_i1-C1A | -0.01 | 0.90 |
|  |  | TRINITY_DN2399_c0_g3_i1-S1A | 0.27 | 0.53 |
|  |  | TRINITY_DN2936_c0_g1_i1-S1A | -1.27 | 0.75 |
|  |  | TRINITY_DN2936_c0_g1_i3-S1A | -0.13 | 0.68 |
|  |  | TRINITY_DN2936_c0_g2_i1-S1A | -0.44 | 0.77 |
|  |  | TRINITY_DN4258_c0_g1_i3-C2A | 0.35 | 0.69 |
|  |  | TRINITY_DN4340_c0_g1_i2-S2A | ND | ND |
|  |  | **TRINITY_DN4396_c0_g3_i1-S3A** | **-2.66** | **0.36** |
|  |  | TRINITY_DN1144_c0_g2_i2-S2A | 0.15 | 0.63 |
|  |  | TRINITY_DN1617_c0_g2_i1-C1A | 0.00 | 1.00 |
|  |  | TRINITY_DN2770_c0_g1_i1-C3A | -0.25 | 0.42 |
|  |  | TRINITY_DN2770_c0_g1_i2-C3A | -0.04 | 0.85 |
|  |  | TRINITY_DN2981_c0_g1_i5-C1A | 0.09 | 0.95 |
|  |  | TRINITY_DN4049_c0_g2_i1-C2A | 1.36 | 0.31 |
|  |  | TRINITY_DN5110_c0_g1_i1-C3A | ND | ND |
|  |  | TRINITY_DN8031_c0_g1_i1-S3A | -0.32 | 0.78 |
| Succinate dehydrogenase | SDH | TRINITY_DN1132_c0_g2_i1-S2A | 0.03 | 0.80 |
|  |  | TRINITY_DN8265_c0_g1_i1-C1A | 0.04 | 0.85 |
|  |  | TRINITY_DN1846_c0_g2_i1-C3A | 0.04 | 0.69 |
| Triose phosphate isomerase | TPI/TIM | TRINITY_DN10622_c0_g1_i1-C1A | -0.11 | 0.70 |
|  |  | TRINITY_DN1087_c0_g1_i1-C2A | 0.20 | 0.46 |
|  |  | TRINITY_DN775_c0_g1_i2-C1A | 0.32 | 0.32 |
| Transaldolase | TAL | TRINITY_DN1327_c0_g2_i1-S1A | 0.04 | 0.77 |
|  |  | TRINITY_DN674_c0_g1_i1-S3A | 0.18 | 0.54 |
| Transketolase | TKT | TRINITY_DN2421_c0_g1_i1-C2A | -0.02 | 0.86 |
|  |  | TRINITY_DN1825_c0_g1_i1-S1A | -0.15 | 0.42 |
|  |  | TRINITY_DN353_c0_g1_i2-S2A | 0.02 | 0.88 |
|  |  | TRINITY_DN7557_c0_g1_i1-C2A | 0.02 | 0.80 |
| Xylose isomerase | XI | TRINITY_DN7515_c0_g1_i1-S2A | 0.11 | 0.04 |
|  |  | TRINITY_DN1555_c0_g1_i1-C2A | 0.04 | 0.57 |
|  |  | TRINITY_DN3461_c0_g1_i1-S1A | -0.01 | 0.98 |
|  |  | TRINITY_DN3956_c0_g1_i2-S3A | -0.03 | 0.93 |
|  |  | TRINITY_DN5292_c0_g1_i3-S3A | -1.21 | 0.59 |
| Xylose redutase | XR | TRINITY_DN6857_c0_g1_i1-C1A | 0.13 | 0.38 |

Note: FC and ND separately indicated foldchange and no detected. The up- and down- regulated DEGs were colored red and green, respectively. The positive and negative numbers indicated up- and down-regulated genes when adding 4.0 g/L taurine.

**Table S3** The gene expression level of MonAzPs biosynthesis pathway for *M. purpureus* treated with taurine.

| **Product** | **Protein** | **Gene** | **Gene locus_tag** | Log_2_Foldchange | *p*_value |
| --- | --- | --- | --- | --- | --- |
| MFS multidrug transporter | QGA67221.1 | MPsGeQ | **TRINITY_DN679_c0_g1_i1-C2A** | **7.96** | **0.00** |
|  |  |  | **TRINITY_DN679_c0_g1_i2-C2A** | **3.64** | **0.01** |
|  |  |  | **TRINITY_DN679_c0_g1_i6-C2A** | **3.70** | **0.13** |
|  |  |  | **TRINITY_DN679_c0_g1_i8-C2A** | **3.34** | **0.37** |
| Hypothetical protein | QGA67222.1 | MPsGeP | **TRINITY_DN2118_c0_g1_i1-C2A** | **5.53** | **0.10** |
|  |  |  | **TRINITY_DN2118_c0_g1_i2-C2A** | **4.30** | **0.01** |
| Deacetylase | QGA67223.1 | MPsGeO | TRINITY_DN2184_c0_g1_i1-S2A | 0.03 | 0.96 |
|  |  |  | TRINITY_DN5235_c0_g1_i1-S3A | 0.06 | 0.83 |
|  |  |  | TRINITY_DN1125_c0_g1_i3-C3A | -0.19 | 0.43 |
|  |  |  | TRINITY_DN1251_c0_g1_i1-C2A | 0.03 | 0.88 |
|  |  |  | TRINITY_DN1251_c0_g1_i3-C2A | -0.04 | 0.92 |
|  |  |  | TRINITY_DN1529_c0_g3_i1-C1A | 1.94 | 0.63 |
|  |  |  | TRINITY_DN1971_c0_g1_i1-C3A | 0.13 | 0.34 |
|  |  |  | TRINITY_DN1767_c0_g2_i1-C2A | -0.04 | 0.75 |
|  |  |  | TRINITY_DN982_c0_g3_i2-C1A | 0.39 | 0.23 |
|  |  |  | TRINITY_DN7725_c0_g1_i1-C2A | 1.07 | 0.10 |
|  |  |  | TRINITY_DN3073_c0_g1_i1-S1A | -0.36 | 0.11 |
|  |  |  | TRINITY_DN3073_c0_g1_i2-S1A | 0.07 | 0.92 |
|  |  |  | TRINITY_DN3775_c0_g1_i2-C3A | -0.12 | 0.43 |
|  |  |  | TRINITY_DN6806_c0_g1_i1-S1A | 0.03 | 0.70 |
|  |  |  | TRINITY_DN1133_c0_g1_i1-S1A | -0.28 | 0.56 |
|  |  |  | TRINITY_DN1430_c0_g1_i1-C2A | 0.07 | 0.81 |
|  |  |  | TRINITY_DN1430_c0_g1_i2-C2A | -0.02 | 0.87 |
|  |  |  | TRINITY_DN34_c0_g1_i1-C2A | 0.51 | 0.17 |
|  |  |  | TRINITY_DN6671_c0_g1_i1-S3A | -0.01 | 0.93 |
|  |  |  | TRINITY_DN1277_c0_g1_i1-S3A | 0.01 | 0.92 |
|  |  |  | TRINITY_DN1767_c2_g2_i1-C2A | 0.07 | 0.81 |
|  |  |  | TRINITY_DN5879_c0_g1_i1-C1A | -0.01 | 0.99 |
|  |  |  | TRINITY_DN608_c0_g1_i1-C2A | 1.01 | 0.14 |
|  |  |  | TRINITY_DN608_c0_g1_i2-C2A | 1.96 | 0.07 |
|  |  |  | TRINITY_DN7965_c0_g1_i1-S1A | -0.01 | 0.91 |
|  |  |  | TRINITY_DN693_c0_g1_i2-S3A | -0.28 | 0.32 |
|  |  |  | TRINITY_DN2564_c1_g1_i1-S1A | 0.04 | 0.83 |
|  |  |  | **TRINITY_DN10365_c0_g1_i1-S1A** | **-3.64** | **0.19** |
|  |  |  | TRINITY_DN9786_c0_g1_i1-C3A | -0.07 | 0.72 |
|  |  |  | TRINITY_DN2960_c0_g1_i1-C2A | 0.13 | 0.27 |
|  |  |  | TRINITY_DN10512_c0_g1_i1-C2A | 0.00 | 0.98 |
|  |  |  | TRINITY_DN1908_c0_g1_i1-S1A | -0.12 | 0.68 |
|  |  |  | TRINITY_DN2806_c0_g1_i1-C3A | 0.06 | 0.60 |
|  |  |  | TRINITY_DN3569_c0_g1_i1-C3A | 0.12 | 0.24 |
|  |  |  | TRINITY_DN731_c0_g1_i2-S2A | -0.02 | 0.91 |
|  |  |  | TRINITY_DN894_c0_g1_i1-C1A | 0.17 | 0.77 |
|  |  |  | TRINITY_DN10010_c0_g1_i1-S3A | 0.04 | 0.81 |
|  |  |  | TRINITY_DN4799_c0_g2_i1-S1A | -0.01 | 0.98 |
|  |  |  | TRINITY_DN7895_c0_g1_i1-S2A | -0.70 | 0.59 |
|  |  |  | TRINITY_DN1767_c2_g1_i1-C2A | 1.89 | 0.64 |
|  |  |  | TRINITY_DN2532_c0_g1_i1-C3A | -0.73 | 0.29 |
|  |  |  | TRINITY_DN369_c0_g1_i2-S3A | -0.58 | 0.47 |
|  |  |  | TRINITY_DN382_c1_g1_i1-S2A | -0.27 | 0.61 |
|  |  |  | TRINITY_DN4547_c0_g1_i2-S2A | 0.63 | 0.30 |
|  |  |  | TRINITY_DN4591_c0_g1_i1-S2A | -1.83 | 0.65 |
|  |  |  | TRINITY_DN946_c0_g1_i1-C2A | 0.09 | 0.43 |
|  |  |  | TRINITY_DN9621_c0_g1_i1-S3A | -1.85 | 0.65 |
| Monooxygenase | QGA67224.1 | MPsGeN | TRINITY_DN3389_c0_g1_i1-S1A | -0.05 | 0.63 |
|  |  |  | TRINITY_DN10819_c0_g1_i1-S2A | 1.27 | 0.41 |
|  |  |  | TRINITY_DN2278_c0_g1_i1-C2A | 0.21 | 0.51 |
|  |  |  | TRINITY_DN2658_c0_g2_i1-S2A | 0.28 | 0.47 |
|  |  |  | **TRINITY_DN3271_c0_g1_i1-C2A** | **5.13** | **0.19** |
|  |  |  | TRINITY_DN3408_c0_g1_i2-S2A | -0.31 | 0.36 |
|  |  |  | TRINITY_DN3574_c0_g1_i1-C2A | 0.76 | 0.55 |
|  |  |  | TRINITY_DN3574_c0_g1_i4-C2A | 0.28 | 0.74 |
|  |  |  | TRINITY_DN3758_c0_g1_i1-S1A | 0.38 | 0.48 |
|  |  |  | TRINITY_DN3853_c0_g1_i1-S3A | 0.04 | 0.83 |
|  |  |  | TRINITY_DN438_c0_g1_i1-C2A | 0.31 | 0.43 |
|  |  |  | TRINITY_DN4780_c0_g1_i1-S2A | -1.91 | 0.63 |
|  |  |  | TRINITY_DN572_c0_g1_i1-C3A | 0.13 | 0.48 |
|  |  |  | TRINITY_DN5815_c0_g1_i1-C3A | -0.45 | 0.68 |
|  |  |  | TRINITY_DN6574_c0_g1_i1-C1A | -0.09 | 0.49 |
|  |  |  | **TRINITY_DN7576_c0_g1_i1-C2A** | **4.43** | **0.00** |
|  |  |  | TRINITY_DN7800_c0_g1_i1-C2A | 0.27 | 0.69 |
|  |  |  | TRINITY_DN8691_c0_g1_i1-S1A | -1.75 | 0.44 |
|  |  |  | TRINITY_DN9761_c0_g1_i1-S1A | -0.69 | 0.78 |
|  |  |  | TRINITY_DN7728_c0_g1_i1-C3A | 0.22 | 0.74 |
|  |  |  | TRINITY_DN2998_c0_g2_i1-C2A | 0.95 | 0.13 |
|  |  |  | TRINITY_DN2674_c0_g1_i2-C3A | -0.05 | 0.77 |
|  |  |  | TRINITY_DN464_c0_g1_i1-S3A | 0.03 | 0.84 |
|  |  |  | TRINITY_DN3070_c0_g2_i1-S2A | 0.09 | 0.95 |
|  |  |  | TRINITY_DN2279_c0_g1_i1-C2A | -0.70 | 0.71 |
|  |  |  | TRINITY_DN2279_c0_g1_i3-C2A | -0.04 | 0.92 |
|  |  |  | **TRINITY_DN10522_c0_g1_i1-S1A** | **-2.40** | **0.55** |
|  |  |  | TRINITY_DN11212_c0_g1_i1-S1A | -1.81 | 0.32 |
|  |  |  | **TRINITY_DN3165_c0_g1_i1-S2A** | **2.07** | **0.42** |
|  |  |  | TRINITY_DN3165_c0_g1_i3-S2A | 0.95 | 0.33 |
|  |  |  | TRINITY_DN3345_c0_g2_i1-C1A | 0.62 | 0.67 |
|  |  |  | TRINITY_DN3951_c0_g1_i1-C3A | 0.30 | 0.78 |
|  |  |  | TRINITY_DN6344_c0_g1_i1-C2A | 0.99 | 0.81 |
|  |  |  | TRINITY_DN653_c0_g1_i1-S3A | -0.44 | 0.06 |
|  |  |  | TRINITY_DN653_c0_g1_i2-S3A | 0.26 | 0.32 |
|  |  |  | TRINITY_DN6848_c0_g1_i1-S2A | ND | ND |
|  |  |  | TRINITY_DN7418_c0_g1_i1-S3A | ND | ND |
|  |  |  | TRINITY_DN7451_c0_g1_i1-S1A | ND | ND |
|  |  |  | TRINITY_DN8543_c0_g1_i1-C1A | ND | ND |
|  |  |  | TRINITY_DN867_c0_g1_i2-C2A | -0.48 | 0.67 |
|  |  |  | TRINITY_DN867_c0_g1_i4-C2A | 0.84 | 0.65 |
| Acyltransferase | QGA67225.1 | MPsGeM | TRINITY_DN1675_c0_g1_i1-S1A | 0.02 | 0.81 |
|  |  |  | TRINITY_DN800_c0_g1_i2-S2A | 0.05 | 0.52 |
|  |  |  | TRINITY_DN800_c0_g1_i3-S2A | -0.17 | 0.51 |
|  |  |  | TRINITY_DN8649_c0_g1_i1-C3A | -0.01 | 0.93 |
|  |  |  | TRINITY_DN994_c0_g1_i1-C2A | 0.13 | 0.54 |
|  |  |  | TRINITY_DN3718_c0_g1_i1-C1A | 0.12 | 0.36 |
|  |  |  | TRINITY_DN5984_c0_g1_i2-S1A | 0.00 | 0.99 |
|  |  |  | TRINITY_DN1089_c0_g1_i1-S3A | -0.09 | 0.49 |
|  |  |  | TRINITY_DN1140_c0_g1_i1-S2A | 0.05 | 0.83 |
|  |  |  | TRINITY_DN1183_c0_g1_i1-C1A | -0.01 | 0.90 |
|  |  |  | TRINITY_DN1258_c0_g1_i1-C2A | 1.27 | 0.00 |
|  |  |  | TRINITY_DN360_c0_g1_i4-S1A | -0.66 | 0.61 |
|  |  |  | TRINITY_DN371_c0_g1_i1-C1A | -0.45 | 0.22 |
|  |  |  | TRINITY_DN371_c0_g1_i2-C1A | -0.02 | 0.93 |
|  |  |  | TRINITY_DN371_c0_g1_i3-C1A | 0.08 | 0.66 |
|  |  |  | TRINITY_DN388_c0_g1_i1-S3A | 0.53 | 0.00 |
|  |  |  | TRINITY_DN388_c0_g1_i2-S3A | -0.22 | 0.08 |
|  |  |  | TRINITY_DN4329_c0_g1_i1-C1A | 0.06 | 0.64 |
|  |  |  | TRINITY_DN830_c0_g1_i2-S2A | -0.75 | 0.10 |
|  |  |  | TRINITY_DN10331_c0_g1_i1-C1A | -0.07 | 0.56 |
|  |  |  | TRINITY_DN10388_c0_g1_i1-C1A | 0.05 | 0.85 |
|  |  |  | TRINITY_DN1457_c2_g1_i1-C2A | 0.47 | 0.28 |
|  |  |  | TRINITY_DN2022_c0_g3_i1-C1A | 0.06 | 0.66 |
|  |  |  | TRINITY_DN2271_c1_g3_i1-S2A | 0.18 | 0.38 |
|  |  |  | TRINITY_DN2672_c0_g1_i1-S3A | 0.19 | 0.36 |
|  |  |  | TRINITY_DN2672_c0_g1_i2-S3A | -0.36 | 0.49 |
|  |  |  | TRINITY_DN2779_c0_g1_i4-C2A | 0.50 | 0.24 |
|  |  |  | TRINITY_DN3186_c0_g2_i3-C3A | 0.00 | 1.00 |
|  |  |  | TRINITY_DN3737_c0_g1_i3-S3A | -0.76 | 0.20 |
|  |  |  | TRINITY_DN5614_c0_g1_i1-C2A | 0.16 | 0.60 |
|  |  |  | TRINITY_DN617_c1_g1_i2-S1A | -0.12 | 0.70 |
|  |  |  | TRINITY_DN7778_c0_g1_i1-S3A | -0.05 | 0.68 |
|  |  |  | TRINITY_DN9193_c0_g1_i1-S3A | -0.03 | 0.88 |
|  |  |  | TRINITY_DN9603_c0_g1_i1-C3A | -0.04 | 0.81 |
|  |  |  | TRINITY_DN1218_c0_g1_i2-C1A | -0.03 | 0.94 |
|  |  |  | TRINITY_DN1218_c0_g1_i3-C1A | -0.73 | 0.23 |
|  |  |  | **TRINITY_DN1218_c0_g1_i4-C1A** | **3.25** | **0.21** |
|  |  |  | TRINITY_DN1218_c0_g1_i5-C1A | -0.04 | 0.91 |
|  |  |  | TRINITY_DN1259_c0_g1_i4-S3A | 0.71 | 0.58 |
|  |  |  | TRINITY_DN1993_c0_g1_i2-S1A | 0.09 | 0.89 |
|  |  |  | TRINITY_DN1993_c0_g1_i4-S1A | -1.86 | 0.62 |
|  |  |  | TRINITY_DN6132_c0_g1_i1-S1A | -0.06 | 0.75 |
|  |  |  | TRINITY_DN2821_c0_g1_i1-S1A | 0.06 | 0.70 |
|  |  |  | TRINITY_DN1337_c0_g2_i1-C1A | -0.04 | 0.94 |
|  |  |  | TRINITY_DN2242_c0_g2_i1-S1A | -0.03 | 0.77 |
|  |  |  | TRINITY_DN2236_c0_g1_i2-S1A | 0.23 | 0.73 |
|  |  |  | TRINITY_DN5478_c0_g1_i2-S3A | 0.06 | 0.87 |
|  |  |  | TRINITY_DN8966_c0_g1_i1-C2A | 0.12 | 0.67 |
|  |  |  | TRINITY_DN9820_c0_g1_i1-C3A | 0.02 | 0.94 |
|  |  |  | TRINITY_DN1704_c0_g1_i1-C3A | 0.01 | 0.93 |
|  |  |  | TRINITY_DN743_c0_g1_i1-C2A | 0.65 | 0.13 |
|  |  |  | TRINITY_DN3877_c0_g1_i1-S1A | -0.06 | 0.50 |
|  |  |  | TRINITY_DN1148_c0_g1_i1-S1A | 0.29 | 0.21 |
|  |  |  | TRINITY_DN1148_c0_g1_i2-S1A | 0.06 | 0.68 |
|  |  |  | TRINITY_DN1324_c0_g1_i1-C1A | -0.12 | 0.56 |
|  |  |  | TRINITY_DN3033_c0_g1_i1-C2A | -0.02 | 0.82 |
|  |  |  | TRINITY_DN3565_c0_g1_i1-C3A | 1.79 | 0.06 |
|  |  |  | TRINITY_DN417_c0_g1_i1-S1A | 0.04 | 0.70 |
|  |  |  | TRINITY_DN417_c0_g1_i2-S1A | 0.31 | 0.48 |
|  |  |  | TRINITY_DN516_c0_g1_i1-C3A | 0.12 | 0.65 |
|  |  |  | TRINITY_DN516_c0_g1_i2-C3A | -0.03 | 0.96 |
|  |  |  | TRINITY_DN1280_c0_g1_i2-S1A | -0.07 | 0.71 |
|  |  |  | TRINITY_DN4897_c0_g1_i1-S3A | -0.18 | 0.46 |
|  |  |  | TRINITY_DN5845_c0_g1_i1-C1A | -0.03 | 0.92 |
|  |  |  | TRINITY_DN7916_c0_g1_i1-C2A | -0.07 | 0.63 |
|  |  |  | TRINITY_DN11212_c0_g1_i1-C2A | 0.03 | 0.99 |
|  |  |  | TRINITY_DN2690_c0_g1_i1-S3A | 0.06 | 0.94 |
| Ankyrin repeat protein | QGA67226.1 | MPsGeL | TRINITY_DN2789_c0_g1_i1-C2A | 0.64 | 0.66 |
|  |  |  | TRINITY_DN2789_c0_g1_i2-C2A | 0.96 | 0.10 |
| FAS subunit beta | QGA67227.1 | MPsGeK | **TRINITY_DN1250_c0_g1_i1-C2A** | **4.16** | **0.01** |
| FAS subunit alpha | QGA67228.1 | MPsGeJ | TRINITY_DN2162_c0_g1_i1-C3A | 0.05 | 0.76 |
|  |  |  | **TRINITY_DN1_c0_g3_i2-C2A** | **4.20** | **0.01** |
|  |  |  | TRINITY_DN2106_c0_g1_i1-S3A | 0.08 | 0.62 |
|  |  |  | TRINITY_DN11195_c0_g1_i1-C2A | ND | ND |
|  |  |  | TRINITY_DN1_c0_g3_i3-C2A | 0.99 | 0.81 |
|  |  |  | TRINITY_DN9120_c0_g1_i1-C2A | ND | ND |
| Transcription factor | QGA67229.1 | MPsGeI | TRINITY_DN10422_c0_g1_i1-C1A | 0.13 | 0.81 |
|  |  |  | TRINITY_DN10565_c0_g1_i1-C1A | 0.72 | 0.22 |
|  |  |  | TRINITY_DN1057_c0_g1_i1-C2A | 0.43 | 0.27 |
|  |  |  | TRINITY_DN1057_c0_g1_i2-C2A | 0.08 | 0.76 |
|  |  |  | TRINITY_DN1057_c0_g1_i3-C2A | -0.05 | 0.86 |
|  |  |  | TRINITY_DN1059_c0_g1_i1-C2A | 0.49 | 0.21 |
|  |  |  | TRINITY_DN10603_c0_g1_i1-C2A | 0.29 | 0.40 |
|  |  |  | TRINITY_DN10849_c0_g1_i1-S1A | 0.15 | 0.37 |
|  |  |  | TRINITY_DN11033_c0_g1_i1-S1A | -0.32 | 0.19 |
|  |  |  | TRINITY_DN1109_c0_g1_i4-S1A | -1.59 | 0.19 |
|  |  |  | TRINITY_DN1109_c0_g1_i5-S1A | ND | ND |
|  |  |  | TRINITY_DN1109_c0_g1_i6-S1A | 0.13 | 0.71 |
|  |  |  | TRINITY_DN1109_c0_g1_i7-S1A | 0.15 | 0.89 |
|  |  |  | TRINITY_DN11134_c0_g1_i1-C2A | 0.11 | 0.84 |
|  |  |  | TRINITY_DN11150_c0_g1_i1-S2A | 0.10 | 0.61 |
|  |  |  | TRINITY_DN11330_c0_g1_i1-S1A | -0.22 | 0.82 |
|  |  |  | TRINITY_DN1137_c0_g1_i1-C2A | 0.35 | 0.30 |
|  |  |  | TRINITY_DN1137_c0_g1_i2-C2A | 0.06 | 0.90 |
|  |  |  | TRINITY_DN1137_c0_g1_i3-C2A | 0.38 | 0.26 |
|  |  |  | TRINITY_DN113_c0_g1_i17-C2A | 0.91 | 0.02 |
|  |  |  | TRINITY_DN113_c0_g1_i25-C2A | 0.13 | 0.81 |
|  |  |  | TRINITY_DN11421_c0_g1_i1-C1A | 0.03 | 0.97 |
|  |  |  | TRINITY_DN1202_c0_g1_i1-C3A | 0.04 | 0.88 |
|  |  |  | TRINITY_DN1216_c0_g1_i2-S3A | -0.29 | 0.39 |
|  |  |  | TRINITY_DN1216_c0_g1_i3-S3A | 0.13 | 0.44 |
|  |  |  | **TRINITY_DN1236_c0_g1_i14-C1A** | **7.14** | **0.00** |
|  |  |  | TRINITY_DN1236_c0_g1_i15-C1A | 0.87 | 0.03 |
|  |  |  | TRINITY_DN1344_c0_g1_i2-C3A | 1.82 | 0.17 |
|  |  |  | TRINITY_DN1344_c0_g1_i3-C3A | -0.17 | 0.52 |
|  |  |  | TRINITY_DN1344_c0_g1_i4-C3A | -0.06 | 0.82 |
|  |  |  | TRINITY_DN1375_c0_g1_i21-S1A | -0.34 | 0.45 |
|  |  |  | TRINITY_DN1375_c0_g1_i4-S1A | -1.59 | 0.42 |
|  |  |  | TRINITY_DN1395_c0_g1_i5-S2A | -0.03 | 0.95 |
|  |  |  | TRINITY_DN1417_c0_g1_i2-S2A | 0.15 | 0.74 |
|  |  |  | TRINITY_DN1417_c0_g1_i3-S2A | 0.77 | 0.11 |
|  |  |  | TRINITY_DN1426_c0_g1_i2-C3A | -0.17 | 0.71 |
|  |  |  | TRINITY_DN1426_c0_g1_i3-C3A | 0.12 | 0.54 |
|  |  |  | TRINITY_DN1426_c0_g1_i4-C3A | 0.04 | 0.92 |
|  |  |  | TRINITY_DN1447_c0_g1_i3-C2A | 0.21 | 0.87 |
|  |  |  | TRINITY_DN1456_c0_g1_i4-C3A | 0.30 | 0.20 |
|  |  |  | TRINITY_DN1473_c0_g1_i2-S3A | -0.16 | 0.67 |
|  |  |  | TRINITY_DN1498_c0_g1_i1-S2A | -0.02 | 0.89 |
|  |  |  | TRINITY_DN1498_c0_g1_i2-S2A | -0.11 | 0.77 |
|  |  |  | TRINITY_DN1557_c0_g1_i16-S2A | ND | ND |
|  |  |  | TRINITY_DN1557_c0_g1_i18-S2A | -0.51 | 0.68 |
|  |  |  | TRINITY_DN1557_c0_g1_i2-S2A | -0.32 | 0.73 |
|  |  |  | TRINITY_DN1557_c0_g1_i21-S2A | 0.12 | 0.94 |
|  |  |  | TRINITY_DN1557_c0_g1_i22-S2A | -1.49 | 0.71 |
|  |  |  | TRINITY_DN1557_c0_g1_i4-S2A | 1.25 | 0.72 |
|  |  |  | TRINITY_DN1597_c0_g1_i3-C3A | 0.91 | 0.82 |
|  |  |  | TRINITY_DN1605_c0_g1_i1-C3A | 0.12 | 0.56 |
|  |  |  | TRINITY_DN1608_c0_g1_i1-C3A | 0.11 | 0.80 |
|  |  |  | TRINITY_DN1608_c0_g1_i2-C3A | 0.14 | 0.72 |
|  |  |  | TRINITY_DN1608_c0_g1_i3-C3A | 0.68 | 0.27 |
|  |  |  | TRINITY_DN1608_c0_g1_i4-C3A | 1.25 | 0.06 |
|  |  |  | TRINITY_DN1636_c0_g1_i1-S1A | -0.17 | 0.14 |
|  |  |  | TRINITY_DN1636_c0_g1_i2-S1A | 0.32 | 0.20 |
|  |  |  | TRINITY_DN1678_c0_g1_i2-S1A | -0.07 | 0.68 |
|  |  |  | TRINITY_DN1678_c0_g1_i3-S1A | 0.03 | 0.93 |
|  |  |  | TRINITY_DN1679_c0_g1_i1-C1A | -0.23 | 0.61 |
|  |  |  | TRINITY_DN1679_c0_g1_i2-C1A | 0.01 | 0.97 |
|  |  |  | TRINITY_DN1681_c0_g1_i1-S1A | -0.33 | 0.36 |
|  |  |  | TRINITY_DN1681_c0_g1_i4-S1A | 0.15 | 0.37 |
|  |  |  | TRINITY_DN1736_c0_g1_i4-C1A | -0.72 | 0.60 |
|  |  |  | TRINITY_DN1792_c1_g1_i1-C2A | 0.56 | 0.08 |
|  |  |  | TRINITY_DN1805_c0_g1_i1-C2A | 1.77 | 0.24 |
|  |  |  | TRINITY_DN1805_c0_g1_i4-C2A | -0.55 | 0.69 |
|  |  |  | TRINITY_DN1805_c0_g1_i5-C2A | -1.00 | 0.79 |
|  |  |  | TRINITY_DN1805_c0_g1_i6-C2A | 1.28 | 0.34 |
|  |  |  | TRINITY_DN1863_c0_g2_i1-S1A | -0.14 | 0.68 |
|  |  |  | TRINITY_DN1863_c0_g2_i3-S1A | -1.47 | 0.03 |
|  |  |  | TRINITY_DN1872_c0_g1_i1-S1A | 0.28 | 0.18 |
|  |  |  | TRINITY_DN1872_c0_g1_i2-S1A | 0.30 | 0.47 |
|  |  |  | TRINITY_DN1904_c0_g1_i4-C1A | -0.28 | 0.07 |
|  |  |  | TRINITY_DN1928_c0_g1_i2-S3A | -0.50 | 0.33 |
|  |  |  | TRINITY_DN1936_c0_g1_i5-S2A | -0.49 | 0.19 |
|  |  |  | TRINITY_DN1942_c0_g1_i2-C1A | 0.15 | 0.58 |
|  |  |  | TRINITY_DN2022_c0_g1_i4-C3A | -0.09 | 0.75 |
|  |  |  | TRINITY_DN2041_c0_g1_i1-S2A | -0.30 | 0.20 |
|  |  |  | TRINITY_DN2069_c0_g1_i1-S1A | -0.19 | 0.25 |
|  |  |  | TRINITY_DN2080_c1_g1_i1-C1A | -0.16 | 0.43 |
|  |  |  | TRINITY_DN2082_c0_g1_i1-C2A | 0.60 | 0.19 |
|  |  |  | TRINITY_DN2082_c0_g1_i3-C2A | 0.03 | 0.89 |
|  |  |  | TRINITY_DN2109_c0_g1_i3-S1A | -1.37 | 0.44 |
|  |  |  | TRINITY_DN2109_c0_g1_i6-S1A | -0.72 | 0.60 |
|  |  |  | TRINITY_DN2116_c0_g1_i2-S1A | 0.12 | 0.79 |
|  |  |  | TRINITY_DN2154_c0_g1_i1-S1A | -1.19 | 0.02 |
|  |  |  | TRINITY_DN2182_c0_g1_i2-S2A | -0.74 | 0.12 |
|  |  |  | TRINITY_DN2192_c0_g1_i1-C2A | 0.77 | 0.33 |
|  |  |  | TRINITY_DN2192_c0_g1_i2-C2A | 0.51 | 0.34 |
|  |  |  | **TRINITY_DN2244_c0_g1_i2-C3A** | **-2.07** | **0.28** |
|  |  |  | TRINITY_DN2244_c0_g1_i5-C3A | 0.69 | 0.57 |
|  |  |  | TRINITY_DN225_c0_g1_i19-S2A | 0.96 | 0.09 |
|  |  |  | TRINITY_DN225_c0_g1_i6-S2A | 0.81 | 0.54 |
|  |  |  | TRINITY_DN225_c0_g1_i7-S2A | 0.68 | 0.20 |
|  |  |  | TRINITY_DN2359_c0_g1_i1-S3A | -0.23 | 0.72 |
|  |  |  | **TRINITY_DN2359_c0_g1_i5-S3A** | **-7.23** | **0.00** |
|  |  |  | TRINITY_DN2363_c0_g1_i1-S2A | ND | ND |
|  |  |  | TRINITY_DN2363_c0_g1_i14-S2A | -0.22 | 0.67 |
|  |  |  | TRINITY_DN2363_c0_g1_i17-S2A | ND | ND |
|  |  |  | TRINITY_DN2363_c0_g1_i4-S2A | 0.16 | 0.60 |
|  |  |  | TRINITY_DN2376_c1_g1_i2-C1A | -0.06 | 0.88 |
|  |  |  | TRINITY_DN2376_c1_g1_i4-C1A | -0.19 | 0.73 |
|  |  |  | TRINITY_DN2456_c0_g1_i1-C2A | 1.13 | 0.19 |
|  |  |  | TRINITY_DN2456_c0_g1_i2-C2A | 0.92 | 0.19 |
|  |  |  | **TRINITY_DN2468_c0_g1_i1-C2A** | **-6.41** | **0.02** |
|  |  |  | TRINITY_DN2468_c0_g1_i4-C2A | 1.61 | 0.00 |
|  |  |  | TRINITY_DN2470_c0_g1_i1-S1A | -0.16 | 0.63 |
|  |  |  | TRINITY_DN2472_c0_g1_i6-C3A | -0.41 | 0.74 |
|  |  |  | TRINITY_DN2474_c0_g1_i2-S1A | 0.51 | 0.26 |
|  |  |  | TRINITY_DN2492_c0_g1_i2-C1A | -0.04 | 0.68 |
|  |  |  | TRINITY_DN2496_c0_g1_i1-C2A | 0.44 | 0.91 |
|  |  |  | TRINITY_DN2496_c0_g1_i4-C2A | 0.83 | 0.66 |
|  |  |  | TRINITY_DN24_c0_g1_i1-S1A | 0.17 | 0.41 |
|  |  |  | TRINITY_DN2533_c0_g1_i3-S2A | -0.72 | 0.57 |
|  |  |  | TRINITY_DN2536_c0_g1_i1-S1A | 0.07 | 0.79 |
|  |  |  | TRINITY_DN2536_c0_g1_i4-S1A | -0.69 | 0.10 |
|  |  |  | TRINITY_DN2556_c1_g1_i1-C2A | 0.18 | 0.80 |
|  |  |  | TRINITY_DN2560_c0_g1_i1-S2A | 0.03 | 0.95 |
|  |  |  | TRINITY_DN2563_c0_g1_i2-S2A | -1.77 | 0.17 |
|  |  |  | TRINITY_DN2651_c0_g1_i1-C1A | 0.12 | 0.36 |
|  |  |  | TRINITY_DN2675_c0_g1_i1-C2A | 0.13 | 0.70 |
|  |  |  | TRINITY_DN2675_c0_g1_i3-C2A | 0.08 | 0.96 |
|  |  |  | TRINITY_DN2676_c0_g2_i1-S1A | -0.14 | 0.48 |
|  |  |  | TRINITY_DN2684_c0_g1_i1-S2A | -0.07 | 0.71 |
|  |  |  | TRINITY_DN2684_c0_g1_i2-S2A | -0.78 | 0.27 |
|  |  |  | TRINITY_DN2686_c0_g1_i1-C1A | -0.14 | 0.53 |
|  |  |  | TRINITY_DN2709_c0_g1_i1-C2A | 0.38 | 0.36 |
|  |  |  | TRINITY_DN2709_c0_g1_i3-C2A | 0.05 | 0.91 |
|  |  |  | TRINITY_DN2710_c0_g1_i1-C1A | -0.07 | 0.42 |
|  |  |  | TRINITY_DN2716_c0_g1_i11-C2A | -0.83 | 0.17 |
|  |  |  | **TRINITY_DN2716_c0_g1_i12-C2A** | **2.37** | **0.23** |
|  |  |  | TRINITY_DN2716_c0_g1_i13-C2A | 0.04 | 0.96 |
|  |  |  | TRINITY_DN2716_c0_g1_i4-C2A | 0.55 | 0.71 |
|  |  |  | TRINITY_DN2716_c0_g1_i9-C2A | 0.99 | 0.28 |
|  |  |  | **TRINITY_DN272_c0_g1_i13-S1A** | **20.85** | **0.00** |
|  |  |  | TRINITY_DN272_c0_g1_i14-S1A | 0.74 | 0.77 |
|  |  |  | **TRINITY_DN272_c0_g1_i20-S1A** | **21.50** | **0.00** |
|  |  |  | TRINITY_DN272_c0_g1_i5-S1A | -0.53 | 0.10 |
|  |  |  | **TRINITY_DN272_c0_g1_i9-S1A** | **4.69** | **0.23** |
|  |  |  | TRINITY_DN2749_c0_g1_i5-S3A | -0.96 | 0.80 |
|  |  |  | TRINITY_DN2796_c1_g2_i1-C2A | 0.55 | 0.47 |
|  |  |  | TRINITY_DN2796_c1_g2_i2-C2A | 1.11 | 0.10 |
|  |  |  | TRINITY_DN2826_c0_g1_i1-C2A | 0.05 | 0.69 |
|  |  |  | TRINITY_DN2831_c0_g1_i1-C2A | 0.40 | 0.37 |
|  |  |  | TRINITY_DN2880_c0_g1_i3-S1A | 0.14 | 0.68 |
|  |  |  | TRINITY_DN2902_c0_g2_i2-S1A | -0.29 | 0.54 |
|  |  |  | TRINITY_DN2909_c0_g1_i2-C3A | 0.13 | 0.77 |
|  |  |  | TRINITY_DN2909_c0_g1_i2-S1A | -0.13 | 0.45 |
|  |  |  | TRINITY_DN2911_c0_g1_i2-S3A | 0.89 | 0.07 |
|  |  |  | TRINITY_DN2911_c0_g1_i3-S3A | -0.31 | 0.05 |
|  |  |  | TRINITY_DN2923_c0_g2_i1-S3A | -0.11 | 0.78 |
|  |  |  | TRINITY_DN2956_c0_g1_i1-C2A | 0.13 | 0.79 |
|  |  |  | TRINITY_DN2956_c0_g1_i2-C2A | -0.32 | 0.33 |
|  |  |  | TRINITY_DN2956_c0_g1_i3-C2A | 0.44 | 0.34 |
|  |  |  | TRINITY_DN2966_c0_g1_i2-S1A | 0.16 | 0.80 |
|  |  |  | TRINITY_DN2977_c0_g1_i2-C1A | 0.09 | 0.91 |
|  |  |  | TRINITY_DN2986_c0_g1_i1-S3A | -0.21 | 0.28 |
|  |  |  | TRINITY_DN2986_c0_g1_i2-S3A | 0.20 | 0.65 |
|  |  |  | TRINITY_DN3002_c0_g1_i1-S3A | -0.53 | 0.23 |
|  |  |  | TRINITY_DN3002_c0_g1_i2-S3A | -0.07 | 0.75 |
|  |  |  | TRINITY_DN3032_c0_g1_i1-S1A | 0.09 | 0.75 |
|  |  |  | TRINITY_DN3072_c0_g5_i1-C2A | 0.13 | 0.79 |
|  |  |  | TRINITY_DN3082_c0_g1_i1-S2A | -0.16 | 0.63 |
|  |  |  | TRINITY_DN3082_c0_g1_i2-S2A | -0.36 | 0.63 |
|  |  |  | TRINITY_DN3133_c0_g1_i1-C1A | -0.04 | 0.73 |
|  |  |  | TRINITY_DN3135_c0_g1_i1-S1A | 0.36 | 0.34 |
|  |  |  | TRINITY_DN3145_c0_g1_i3-S1A | -0.43 | 0.12 |
|  |  |  | TRINITY_DN331_c0_g1_i12-S3A | ND | ND |
|  |  |  | TRINITY_DN3359_c0_g1_i2-S3A | 0.41 | 0.38 |
|  |  |  | TRINITY_DN3419_c0_g1_i1-C1A | 0.18 | 0.29 |
|  |  |  | TRINITY_DN3426_c0_g1_i1-S1A | 0.32 | 0.70 |
|  |  |  | TRINITY_DN3426_c0_g1_i2-S1A | -0.83 | 0.14 |
|  |  |  | TRINITY_DN3467_c0_g1_i1-C1A | 0.02 | 0.94 |
|  |  |  | TRINITY_DN3486_c0_g1_i2-C2A | -0.38 | 0.54 |
|  |  |  | TRINITY_DN349_c0_g1_i5-C1A | -0.09 | 0.82 |
|  |  |  | TRINITY_DN3501_c0_g1_i1-S2A | -0.01 | 0.97 |
|  |  |  | TRINITY_DN3501_c0_g1_i2-S2A | -0.52 | 0.42 |
|  |  |  | TRINITY_DN3513_c0_g1_i2-S2A | 0.82 | 0.18 |
|  |  |  | TRINITY_DN3514_c0_g1_i1-S2A | -1.27 | 0.10 |
|  |  |  | **TRINITY_DN3550_c0_g1_i1-S3A** | **-2.16** | **0.21** |
|  |  |  | TRINITY_DN3570_c0_g1_i1-C2A | -0.06 | 0.52 |
|  |  |  | TRINITY_DN35_c0_g1_i13-C2A | 1.01 | 0.71 |
|  |  |  | TRINITY_DN35_c0_g1_i17-C2A | 1.36 | 0.11 |
|  |  |  | TRINITY_DN35_c0_g1_i20-C2A | 0.78 | 0.33 |
|  |  |  | **TRINITY_DN35_c0_g1_i7-C2A** | **20.99** | **0.00** |
|  |  |  | TRINITY_DN3678_c0_g1_i2-S1A | -0.14 | 0.73 |
|  |  |  | TRINITY_DN3678_c0_g1_i4-S1A | 0.07 | 0.84 |
|  |  |  | TRINITY_DN3678_c0_g1_i5-S1A | -0.08 | 0.66 |
|  |  |  | TRINITY_DN3696_c0_g1_i1-C2A | 0.34 | 0.44 |
|  |  |  | TRINITY_DN3710_c0_g1_i1-S3A | 0.13 | 0.66 |
|  |  |  | TRINITY_DN3716_c0_g2_i1-C2A | 0.27 | 0.72 |
|  |  |  | **TRINITY_DN3869_c0_g4_i1-S1A** | **-4.61** | **0.17** |
|  |  |  | TRINITY_DN3897_c0_g3_i1-S1A | -1.36 | 0.47 |
|  |  |  | TRINITY_DN3900_c0_g3_i1-S3A | -1.57 | 0.40 |
|  |  |  | TRINITY_DN3914_c0_g1_i2-S2A | -0.16 | 0.74 |
|  |  |  | TRINITY_DN3914_c0_g1_i4-S2A | -0.43 | 0.27 |
|  |  |  | TRINITY_DN3920_c0_g1_i1-S2A | -0.72 | 0.23 |
|  |  |  | TRINITY_DN3983_c0_g2_i1-C2A | 1.08 | 0.31 |
|  |  |  | TRINITY_DN3983_c0_g2_i2-C2A | 0.90 | 0.39 |
|  |  |  | TRINITY_DN39_c0_g1_i12-C3A | -0.62 | 0.16 |
|  |  |  | TRINITY_DN39_c0_g1_i13-C3A | 0.31 | 0.62 |
|  |  |  | TRINITY_DN39_c0_g1_i16-C3A | 0.18 | 0.31 |
|  |  |  | TRINITY_DN4026_c0_g1_i1-C2A | 0.06 | 0.63 |
|  |  |  | TRINITY_DN4030_c0_g2_i1-C2A | -0.08 | 0.94 |
|  |  |  | TRINITY_DN4036_c0_g1_i2-C2A | 0.10 | 0.81 |
|  |  |  | TRINITY_DN4067_c0_g1_i1-S3A | 0.18 | 0.54 |
|  |  |  | TRINITY_DN406_c0_g1_i5-C1A | 0.22 | 0.41 |
|  |  |  | TRINITY_DN4139_c0_g2_i1-C1A | 0.53 | 0.78 |
|  |  |  | TRINITY_DN4152_c0_g4_i1-C2A | 0.25 | 0.89 |
|  |  |  | TRINITY_DN4207_c0_g1_i1-S1A | 0.02 | 0.85 |
|  |  |  | TRINITY_DN4242_c0_g4_i1-C1A | -0.46 | 0.76 |
|  |  |  | TRINITY_DN4408_c0_g2_i1-S1A | -0.19 | 0.64 |
|  |  |  | TRINITY_DN4415_c0_g2_i1-S1A | -0.07 | 0.88 |
|  |  |  | **TRINITY_DN4438_c0_g2_i1-S3A** | **-5.91** | **0.00** |
|  |  |  | TRINITY_DN4438_c0_g2_i2-S3A | 1.19 | 0.52 |
|  |  |  | TRINITY_DN4469_c0_g1_i1-C1A | 0.01 | 0.92 |
|  |  |  | TRINITY_DN448_c0_g1_i1-C2A | 0.73 | 0.17 |
|  |  |  | TRINITY_DN448_c0_g1_i2-C2A | 0.41 | 0.65 |
|  |  |  | TRINITY_DN448_c0_g1_i3-C2A | 0.18 | 0.49 |
|  |  |  | TRINITY_DN448_c0_g1_i4-C2A | 0.09 | 0.88 |
|  |  |  | TRINITY_DN4490_c0_g2_i2-C2A | -0.87 | 0.83 |
|  |  |  | TRINITY_DN4490_c0_g2_i3-C2A | 1.06 | 0.16 |
|  |  |  | TRINITY_DN4493_c1_g1_i1-S2A | -0.16 | 0.94 |
|  |  |  | TRINITY_DN4499_c1_g1_i1-S2A | -0.47 | 0.75 |
|  |  |  | TRINITY_DN4550_c0_g1_i1-S1A | -0.03 | 0.89 |
|  |  |  | TRINITY_DN4581_c0_g2_i1-S2A | -0.07 | 0.87 |
|  |  |  | TRINITY_DN45_c0_g1_i2-C2A | -0.01 | 0.96 |
|  |  |  | TRINITY_DN45_c0_g1_i3-C2A | 0.37 | 0.33 |
|  |  |  | TRINITY_DN4699_c0_g1_i2-C1A | 1.05 | 0.40 |
|  |  |  | TRINITY_DN4758_c0_g1_i2-C2A | 0.22 | 0.65 |
|  |  |  | TRINITY_DN4876_c0_g2_i1-C1A | 0.36 | 0.83 |
|  |  |  | TRINITY_DN4884_c0_g1_i1-C2A | 0.59 | 0.48 |
|  |  |  | TRINITY_DN4941_c0_g2_i1-S1A | -0.38 | 0.45 |
|  |  |  | TRINITY_DN5031_c0_g1_i2-C1A | 0.41 | 0.29 |
|  |  |  | TRINITY_DN506_c0_g1_i3-C3A | 0.44 | 0.75 |
|  |  |  | TRINITY_DN506_c0_g1_i8-C3A | -1.36 | 0.29 |
|  |  |  | TRINITY_DN5092_c0_g2_i1-C1A | 0.17 | 0.72 |
|  |  |  | TRINITY_DN5105_c0_g2_i1-S2A | -1.58 | 0.61 |
|  |  |  | TRINITY_DN5164_c0_g1_i1-S3A | 0.09 | 0.67 |
|  |  |  | TRINITY_DN5176_c0_g1_i1-S3A | -0.34 | 0.45 |
|  |  |  | TRINITY_DN5181_c0_g1_i1-C3A | -0.01 | 0.97 |
|  |  |  | TRINITY_DN5188_c0_g1_i1-C3A | 0.30 | 0.85 |
|  |  |  | TRINITY_DN5195_c0_g2_i1-S1A | 0.33 | 0.91 |
|  |  |  | TRINITY_DN5196_c0_g1_i1-C2A | 0.10 | 0.77 |
|  |  |  | **TRINITY_DN5238_c0_g1_i1-C2A** | **2.25** | **0.36** |
|  |  |  | TRINITY_DN5241_c0_g2_i1-C2A | 0.21 | 0.75 |
|  |  |  | TRINITY_DN5268_c0_g1_i1-C3A | -0.01 | 0.99 |
|  |  |  | TRINITY_DN5283_c0_g1_i1-S3A | -0.02 | 0.85 |
|  |  |  | TRINITY_DN5351_c0_g1_i1-S2A | 0.03 | 0.90 |
|  |  |  | TRINITY_DN5361_c0_g1_i1-C2A | 0.64 | 0.38 |
|  |  |  | TRINITY_DN53_c0_g1_i10-S3A | 0.99 | 0.44 |
|  |  |  | TRINITY_DN5485_c0_g1_i1-S2A | 0.14 | 0.52 |
|  |  |  | TRINITY_DN5485_c0_g3_i1-C2A | 1.93 | 0.63 |
|  |  |  | TRINITY_DN5589_c0_g1_i1-C2A | 0.56 | 0.29 |
|  |  |  | TRINITY_DN5694_c0_g1_i1-S2A | -0.45 | 0.72 |
|  |  |  | TRINITY_DN5728_c0_g1_i1-C2A | -0.06 | 0.73 |
|  |  |  | TRINITY_DN5751_c0_g1_i1-S1A | -0.13 | 0.37 |
|  |  |  | TRINITY_DN5791_c0_g1_i1-S3A | 0.13 | 0.48 |
|  |  |  | TRINITY_DN5871_c0_g1_i1-C3A | 0.06 | 0.82 |
|  |  |  | TRINITY_DN5896_c0_g1_i1-S1A | 0.10 | 0.83 |
|  |  |  | TRINITY_DN5896_c0_g1_i2-S1A | -0.40 | 0.06 |
|  |  |  | TRINITY_DN605_c0_g1_i2-C3A | 0.03 | 0.72 |
|  |  |  | TRINITY_DN632_c0_g1_i2-C2A | 0.09 | 0.56 |
|  |  |  | TRINITY_DN632_c0_g1_i3-C2A | 0.45 | 0.26 |
|  |  |  | TRINITY_DN6354_c0_g1_i1-S2A | -0.31 | 0.43 |
|  |  |  | TRINITY_DN6659_c0_g1_i1-C2A | 0.07 | 0.46 |
|  |  |  | TRINITY_DN6677_c0_g1_i1-S1A | -0.10 | 0.59 |
|  |  |  | TRINITY_DN671_c0_g1_i5-C2A | 0.02 | 0.95 |
|  |  |  | TRINITY_DN685_c0_g1_i1-C2A | -0.01 | 0.96 |
|  |  |  | TRINITY_DN685_c0_g1_i2-C2A | -0.13 | 0.37 |
|  |  |  | TRINITY_DN699_c0_g1_i1-S3A | 0.39 | 0.46 |
|  |  |  | TRINITY_DN699_c0_g1_i2-S3A | -0.02 | 0.95 |
|  |  |  | TRINITY_DN699_c0_g1_i3-S3A | -0.53 | 0.16 |
|  |  |  | TRINITY_DN699_c0_g1_i4-S3A | -0.51 | 0.33 |
|  |  |  | TRINITY_DN7478_c0_g1_i1-C3A | 0.16 | 0.57 |
|  |  |  | TRINITY_DN7485_c0_g1_i1-C2A | -0.18 | 0.21 |
|  |  |  | TRINITY_DN7504_c0_g1_i1-S3A | -1.88 | 0.57 |
|  |  |  | TRINITY_DN7676_c0_g1_i1-S3A | 0.34 | 0.57 |
|  |  |  | TRINITY_DN7745_c0_g1_i1-S1A | -0.13 | 0.76 |
|  |  |  | TRINITY_DN7860_c0_g1_i1-S1A | 0.13 | 0.56 |
|  |  |  | TRINITY_DN8161_c0_g1_i1-C2A | 0.95 | 0.22 |
|  |  |  | TRINITY_DN8312_c0_g1_i1-C2A | 1.64 | 0.59 |
|  |  |  | TRINITY_DN841_c0_g1_i6-C3A | 1.41 | 0.37 |
|  |  |  | TRINITY_DN841_c0_g1_i8-C3A | 0.02 | 0.92 |
|  |  |  | TRINITY_DN8861_c0_g1_i1-C3A | 0.08 | 0.93 |
|  |  |  | TRINITY_DN88_c0_g1_i10-C1A | 1.45 | 0.61 |
|  |  |  | TRINITY_DN88_c0_g3_i1-C1A | -0.23 | 0.45 |
|  |  |  | TRINITY_DN9215_c0_g1_i1-S3A | 0.03 | 0.82 |
|  |  |  | TRINITY_DN923_c0_g1_i1-C2A | 0.14 | 0.76 |
|  |  |  | TRINITY_DN923_c0_g1_i2-C2A | 0.09 | 0.73 |
|  |  |  | TRINITY_DN923_c0_g1_i4-C2A | 0.34 | 0.52 |
|  |  |  | TRINITY_DN923_c0_g1_i5-C2A | -0.08 | 0.67 |
|  |  |  | TRINITY_DN9574_c0_g1_i1-C2A | 0.33 | 0.26 |
|  |  |  | TRINITY_DN9577_c0_g1_i1-C3A | 0.21 | 0.53 |
|  |  |  | TRINITY_DN9607_c0_g1_i1-C3A | 0.03 | 0.82 |
|  |  |  | TRINITY_DN9619_c0_g1_i1-S2A | -0.04 | 0.85 |
|  |  |  | TRINITY_DN9724_c0_g1_i1-S2A | -0.04 | 0.86 |
|  |  |  | TRINITY_DN976_c0_g1_i1-S3A | 0.15 | 0.63 |
|  |  |  | TRINITY_DN976_c0_g1_i6-S3A | -0.57 | 0.16 |
|  |  |  | TRINITY_DN982_c0_g1_i2-S3A | 0.23 | 0.93 |
|  |  |  | TRINITY_DN982_c0_g1_i6-S3A | 0.14 | 0.58 |
| Enoyl reductase | QGA67230.1 | MPsGeH | **TRINITY_DN4418_c0_g1_i1-C2A** | **3.42** | **0.02** |
| Serine hydrolase | QGA67231.1 | MPsGeG | TRINITY_DN8935_c0_g1_i1-C1A | 0.12 | 0.49 |
|  |  |  | TRINITY_DN1074_c0_g1_i1-C2A | -0.02 | 0.88 |
|  |  |  | TRINITY_DN2319_c0_g1_i1-S2A | 0.48 | 0.71 |
|  |  |  | **TRINITY_DN2481_c0_g1_i2-C2A** | **4.09** | **0.01** |
|  |  |  | TRINITY_DN2481_c0_g1_i5-C2A | 0.64 | 0.48 |
|  |  |  | TRINITY_DN2481_c0_g1_i8-C2A | 0.25 | 0.39 |
|  |  |  | TRINITY_DN2481_c0_g1_i9-C2A | 1.33 | 0.34 |
|  |  |  | TRINITY_DN3778_c0_g1_i3-S3A | -0.10 | 0.70 |
|  |  |  | **TRINITY_DN4092_c0_g2_i1-S2A** | **-2.44** | **0.54** |
|  |  |  | TRINITY_DN726_c1_g1_i1-S1A | 0.09 | 0.59 |
|  |  |  | TRINITY_DN9614_c0_g1_i1-S3A | -0.55 | 0.83 |
| Oxidoreductase | QGA67232.1 | MPsGeF | **TRINITY_DN9652_c0_g1_i1-C2A** | **3.76** | **0.04** |
| Oxidoreductase | QGA67232.1 | MPsGeF | **TRINITY_DN7801_c0_g1_i1-C2A** | **5.01** | **0.00** |
| Oxidoreductase | QGA67233.1 | MPsGeE | **TRINITY_DN9652_c0_g2_i1-C2A** | **4.85** | **0.00** |
| Acyltransferase | QGA67234.1 | MPsGeD | **TRINITY_DN7828_c0_g1_i1-C2A** | **3.99** | **0.01** |
| C-11-ketoreductase | QGA67235.1 | MPsGeC | **TRINITY_DN2796_c1_g1_i2-C2A** | **23.30** | **0.00** |
| Transcription factor | QGA67236.1 | MPsGeB | **TRINITY_DN9874_c0_g1_i1-C2A** | **2.17** | **0.09** |
| NR-PKS | QGA67237.1 | MPsGeA | **TRINITY_DN2093_c0_g1_i1-C2A** | **4.16** | **0.01** |
|  | QGA67237.1 |  | TRINITY_DN2093_c0_g1_i2-C2A | 1.99 | 0.17 |

**Table S4** The 169 significant DEGs for *M. purpureus* treated with taurine.

| **Product** | **Gene** | **Log_2_FC** | ***p*_value** |
| --- | --- | --- | --- |
| 15-O-acetyltransferase Tri3 | TRINITY_DN2118_c0_g1_i2-C2A | 4.30 | 0.01 |
| AAA domain | TRINITY_DN356_c0_g1_i8-S1A | -5.30 | 0.00 |
|  | TRINITY_DN356_c0_g1_i10-S1A | 4.45 | 0.00 |
| ABC transporter | TRINITY_DN2401_c0_g2_i2-S3A | 7.25 | 0.01 |
| Acetamidase/Formamidase family | TRINITY_DN684_c0_g1_i4-C2A | 2.29 | 0.03 |
| Adenoviral core protein VII | TRINITY_DN830_c0_g2_i3-C2A | 9.43 | 0.00 |
|  | TRINITY_DN520_c1_g1_i5-C1A | 2.60 | 0.01 |
| Aldo/keto reductase family | TRINITY_DN9652_c0_g2_i1-C2A | 4.85 | 0.00 |
| Amino acid permease | TRINITY_DN53_c0_g1_i1-S3A | 8.92 | 0.00 |
|  | TRINITY_DN2675_c0_g2_i2-S1A | -6.34 | 0.01 |
| Ammonium Transporter Family | TRINITY_DN1038_c0_g1_i23-S3A | -5.98 | 0.03 |
| AMP-binding enzyme | TRINITY_DN2475_c0_g1_i4-S1A | 6.85 | 0.01 |
|  | TRINITY_DN69_c0_g2_i1-C2A | 2.37 | 0.05 |
|  | TRINITY_DN1861_c0_g1_i1-S3A | 5.23 | 0.00 |
| ATPase family associated with various cellular activities (AAA) | TRINITY_DN3764_c0_g1_i1-S3A | -2.66 | 0.01 |
| Bacteriorhodopsin-like protein | TRINITY_DN1563_c0_g1_i1-C2A | 4.75 | 0.04 |
| BAR domain | TRINITY_DN104_c0_g1_i2-C2A | 21.66 | 0.00 |
| Beta-ketoacyl synthase, N-terminal domain | TRINITY_DN2093_c0_g1_i1-C2A | 4.16 | 0.01 |
| Beta-lactamase | TRINITY_DN4850_c0_g1_i4-C2A | 3.50 | 0.00 |
| BTB/POZ domain | TRINITY_DN492_c0_g1_i2-C2A | 21.38 | 0.00 |
| Carbohydrate phosphorylase | TRINITY_DN1820_c0_g1_i1-S2A | 4.18 | 0.01 |
| Cation efflux family | TRINITY_DN2573_c0_g1_i1-S2A | -5.24 | 0.01 |
| Cation transport ATPase (P-type) | TRINITY_DN5267_c0_g1_i1-S3A | -2.05 | 0.01 |
| CENP-Q, a CENPA-CAD centromere complex subunit | TRINITY_DN563_c0_g1_i4-C2A | 2.43 | 0.05 |
| CO dehydrogenase beta subunit/acetyl-CoA synthase epsilon subunit | TRINITY_DN1698_c0_g1_i2-C2A | 2.62 | 0.01 |
| Collagen triple helix repeat (20 copies) | TRINITY_DN139_c0_g1_i11-S2A | 2.19 | 0.04 |
| Commissureless | TRINITY_DN235_c0_g1_i8-C2A | 7.57 | 0.00 |
| Cytochrome P450 | TRINITY_DN8651_c0_g1_i1-C2A | 2.09 | 0.02 |
| D-glutamate cyclase | TRINITY_DN6636_c0_g1_i1-C2A | 2.62 | 0.02 |
| Domain of unknown function | TRINITY_DN200_c0_g1_i1-S2A | 3.36 | 0.02 |
| Domain of unknown function (DUF1729) | TRINITY_DN1250_c0_g1_i1-C2A | 4.16 | 0.01 |
|  | TRINITY_DN594_c0_g1_i3-C3A | 2.03 | 0.00 |
|  | TRINITY_DN532_c1_g1_i6-S3A | -7.49 | 0.00 |
|  | TRINITY_DN87_c0_g1_i1-S3A | 3.18 | 0.02 |
|  | TRINITY_DN9976_c0_g1_i1-C2A | 3.60 | 0.04 |
| Dual specificity phosphatase, catalytic domain | TRINITY_DN301_c0_g1_i2-C2A | 2.62 | 0.01 |
| Ecdysteroid kinase-like family | TRINITY_DN5172_c0_g1_i1-S3A | -6.65 | 0.00 |
| Elongation factor Tu GTP binding domain | TRINITY_DN292_c0_g1_i1-C3A | 6.53 | 0.00 |
|  | TRINITY_DN266_c0_g1_i5-C2A | -21.45 | 0.00 |
| Enoyl-(Acyl carrier protein) reductase | TRINITY_DN1103_c0_g1_i1-C2A | 2.38 | 0.01 |
| Ergosterol biosynthesis ERG4/ERG24 family | TRINITY_DN2131_c0_g1_i1-C2A | 6.55 | 0.03 |
| FAD binding domain | TRINITY_DN7576_c0_g1_i1-C2A | 4.43 | 0.00 |
|  | TRINITY_DN2696_c0_g1_i1-S2A | -4.11 | 0.00 |
| Fatty acid synthase subunit alpha Acyl carrier domain | TRINITY_DN1_c0_g3_i2-C2A | 4.20 | 0.01 |
| Ferric reductase NAD binding domain | TRINITY_DN360_c0_g1_i3-C3A | -2.56 | 0.03 |
| Flavin containing amine oxidoreductase | TRINITY_DN1990_c0_g1_i3-S3A | -6.00 | 0.00 |
| FMN-dependent dehydrogenase | TRINITY_DN5478_c0_g2_i1-S1A | -5.64 | 0.00 |
| Fungal specific transcription factor domain | TRINITY_DN272_c0_g1_i13-S1A | 20.85 | 0.00 |
|  | TRINITY_DN2468_c0_g1_i1-C2A | -6.41 | 0.02 |
|  | TRINITY_DN1236_c0_g1_i14-C1A | 7.14 | 0.00 |
|  | TRINITY_DN272_c0_g1_i20-S1A | 21.50 | 0.00 |
|  | TRINITY_DN35_c0_g1_i7-C2A | 20.99 | 0.00 |
|  | TRINITY_DN2359_c0_g1_i5-S3A | -7.23 | 0.00 |
|  | TRINITY_DN4438_c0_g2_i1-S3A | -5.91 | 0.00 |
| GAT domain | TRINITY_DN565_c0_g2_i1-C2A | 4.94 | 0.00 |
| Glycosyl hydrolase family 3 C-terminal domain | TRINITY_DN4299_c0_g1_i1-C1A | 5.34 | 0.00 |
| Glycosyl hydrolases family 18 | TRINITY_DN134_c0_g1_i7-S3A | -6.89 | 0.01 |
| GMC oxidoreductase | TRINITY_DN1699_c1_g2_i2-C2A | 3.54 | 0.00 |
| GPI ethanolamine phosphate transferase membrane region | TRINITY_DN952_c0_g1_i4-S2A | -5.99 | 0.00 |
| haloacid dehalogenase-like hydrolase | TRINITY_DN9022_c0_g1_i1-C3A | 4.88 | 0.03 |
| HIRA B motif | TRINITY_DN4719_c0_g1_i3-S1A | -5.30 | 0.01 |
| Indigoidine synthase A like protein | TRINITY_DN711_c0_g1_i6-C2A | 8.98 | 0.00 |
| Integrase zinc binding domain | TRINITY_DN675_c0_g1_i6-S2A | 6.16 | 0.02 |
|  | TRINITY_DN675_c0_g1_i8-S2A | 6.50 | 0.00 |
| Iron/manganese superoxide dismutases, C-terminal domain | TRINITY_DN3159_c0_g1_i2-C1A | 4.00 | 0.00 |
|  | TRINITY_DN1092_c0_g1_i2-C2A | 2.68 | 0.01 |
| Late cornified envelope protein 6A family | TRINITY_DN1_c1_g1_i3-C2A | 3.70 | 0.02 |
|  | TRINITY_DN4583_c0_g1_i1-C2A | 2.69 | 0.04 |
| Major Facilitator Superfamily | TRINITY_DN326_c0_g1_i1-C2A | 4.72 | 0.02 |
|  | TRINITY_DN2036_c0_g1_i1-S2A | -4.60 | 0.00 |
|  | TRINITY_DN679_c0_g1_i1-C2A | 7.96 | 0.00 |
|  | TRINITY_DN1569_c0_g1_i4-C2A | -2.21 | 0.00 |
|  | TRINITY_DN2036_c0_g1_i5-S2A | -4.70 | 0.00 |
|  | TRINITY_DN679_c0_g1_i2-C2A | 3.64 | 0.01 |
|  | TRINITY_DN679_c0_g1_i3-C2A | 3.77 | 0.01 |
|  | TRINITY_DN679_c0_g1_i4-C2A | 21.12 | 0.00 |
|  | TRINITY_DN679_c0_g1_i7-C2A | 22.02 | 0.00 |
|  | TRINITY_DN326_c0_g1_i5-C2A | 7.17 | 0.01 |
|  | TRINITY_DN326_c0_g1_i4-C2A | 21.24 | 0.00 |
| Methyltransferase domain | TRINITY_DN2602_c0_g1_i1-S2A | -4.74 | 0.03 |
| Microtubule-associated protein 70 | TRINITY_DN4752_c0_g1_i4-C2A | 3.46 | 0.04 |
| Mitochondrial protein from FMP27 | TRINITY_DN2224_c0_g3_i1-S3A | -3.77 | 0.04 |
| Molybdate transporter of MFS superfamily | TRINITY_DN3272_c0_g1_i1-C2A | 3.32 | 0.01 |
| MYND finger | TRINITY_DN531_c0_g1_i5-S1A | 20.82 | 0.00 |
| NAD(P)-binding Rossmann-like domain | TRINITY_DN9652_c0_g1_i1-C2A | 3.76 | 0.04 |
| NAD(P)H-binding | TRINITY_DN5093_c0_g2_i1-C2A | 4.01 | 0.03 |
| NDT80 / PhoG like DNA-binding family | TRINITY_DN4011_c0_g1_i2-C3A | 7.34 | 0.00 |
| NIF3 (NGG1p interacting factor 3) | TRINITY_DN246_c0_g1_i2-S3A | 6.92 | 0.01 |
| Pectate lyase superfamily protein | TRINITY_DN416_c0_g1_i11-S3A | -22.37 | 0.00 |
| Peptidase family M3 | TRINITY_DN3911_c0_g1_i3-S1A | 4.55 | 0.00 |
| Permease for cytosine/purines, uracil, thiamine, allantoin | TRINITY_DN1605_c0_g1_i1-C2A | 2.19 | 0.01 |
| Phosphatidylethanolamine-binding protein | TRINITY_DN4854_c0_g1_i1-C2A | 2.26 | 0.04 |
| Phthiocerol/phthiodiolone dimycocerosyl transferase C-terminus | TRINITY_DN7655_c0_g1_i1-C2A | 4.17 | 0.01 |
| PLD-like domain | TRINITY_DN1995_c0_g1_i3-C2A | 2.16 | 0.01 |
| Polynucleotide kinase 3 phosphatase | TRINITY_DN710_c0_g1_i15-S1A | 20.92 | 0.00 |
| POT family | TRINITY_DN8754_c0_g1_i1-C2A | 2.71 | 0.04 |
| Prephenate dehydrogenase, dimerization domain | TRINITY_DN491_c0_g1_i3-C2A | 2.78 | 0.01 |
| Protein kinase domain | TRINITY_DN692_c0_g1_i3-C3A | 5.74 | 0.00 |
| Protein of unknown function (DUF1387) | TRINITY_DN26_c0_g1_i8-C1A | -20.18 | 0.00 |
|  | TRINITY_DN977_c0_g1_i1-C2A | 2.27 | 0.03 |
|  | TRINITY_DN1588_c0_g1_i2-S3A | -21.41 | 0.00 |
|  | TRINITY_DN96_c0_g1_i5-C1A | -6.17 | 0.02 |
|  | TRINITY_DN131_c0_g1_i5-S2A | 7.11 | 0.00 |
|  | TRINITY_DN4703_c0_g1_i1-C2A | 2.49 | 0.02 |
|  | TRINITY_DN1301_c0_g1_i1-C1A | 2.56 | 0.01 |
| Protein phosphatase 2C | TRINITY_DN3676_c0_g1_i1-C1A | 3.19 | 0.00 |
| Pyoverdine/dityrosine biosynthesis protein | TRINITY_DN10096_c0_g1_i1-C2A | 4.08 | 0.00 |
| RNA methyltransferase | TRINITY_DN1995_c0_g2_i9-C2A | 5.14 | 0.00 |
| Serine hydrolase (FSH1) | TRINITY_DN4418_c0_g1_i1-C2A | 3.42 | 0.02 |
|  | TRINITY_DN7801_c0_g1_i1-C2A | 5.01 | 0.00 |
|  | TRINITY_DN2481_c0_g1_i2-C2A | 4.09 | 0.01 |
| Short chain dehydrogenase | TRINITY_DN2796_c1_g1_i2-C2A | 23.30 | 0.00 |
|  | TRINITY_DN2796_c1_g1_i3-C2A | 4.00 | 0.01 |
| Speriolin C-terminus | TRINITY_DN1883_c0_g1_i5-C2A | 2.33 | 0.01 |
|  | TRINITY_DN1883_c0_g1_i3-C2A | 2.46 | 0.00 |
| Sugar (and other) transporter | TRINITY_DN1926_c0_g1_i2-C2A | 2.06 | 0.01 |
| Sulfatase | TRINITY_DN1580_c0_g1_i2-S2A | 5.32 | 0.02 |
| Taurine catabolism dioxygenase TauD, TfdA family | TRINITY_DN5754_c0_g1_i1-C2A | 3.06 | 0.02 |
| Thiamin pyrophosphokinase, vitamin B1 binding domain | TRINITY_DN278_c0_g1_i1-C2A | 21.12 | 0.00 |
| TMEM214, C-terminal, caspase 4 activator | TRINITY_DN2594_c0_g1_i4-C2A | 8.79 | 0.02 |
| TNP1/EN/SPM transposase | TRINITY_DN755_c0_g1_i4-C2A | -6.65 | 0.01 |
| Transferase family | TRINITY_DN7828_c0_g1_i1-C2A | 3.99 | 0.01 |
| tRNA synthetases class I (W and Y) | TRINITY_DN256_c0_g1_i5-S1A | 3.47 | 0.00 |
| Type III secretion system lipoprotein chaperone (YscW) | TRINITY_DN2603_c0_g1_i4-C2A | 6.88 | 0.00 |
|  | TRINITY_DN4708_c0_g1_i3-S3A | -5.70 | 0.05 |
| Vacuole effluxer Atg22 like | TRINITY_DN10570_c0_g1_i1-C2A | 5.27 | 0.00 |
| VirK protein | TRINITY_DN1920_c0_g1_i3-S2A | -4.24 | 0.00 |
|  | TRINITY_DN1920_c0_g1_i1-S2A | 4.92 | 0.00 |
| Zinc knuckle | TRINITY_DN1343_c0_g1_i5-C2A | 7.69 | 0.00 |
| Zinc-binding dehydrogenase | TRINITY_DN256_c0_g1_i2-C2A | 2.12 | 0.02 |
| Zinc-finger double domain | TRINITY_DN3445_c0_g1_i1-C3A | 5.52 | 0.01 |
| ZIP Zinc transporter | TRINITY_DN3962_c0_g1_i1-S2A | -3.46 | 0.02 |


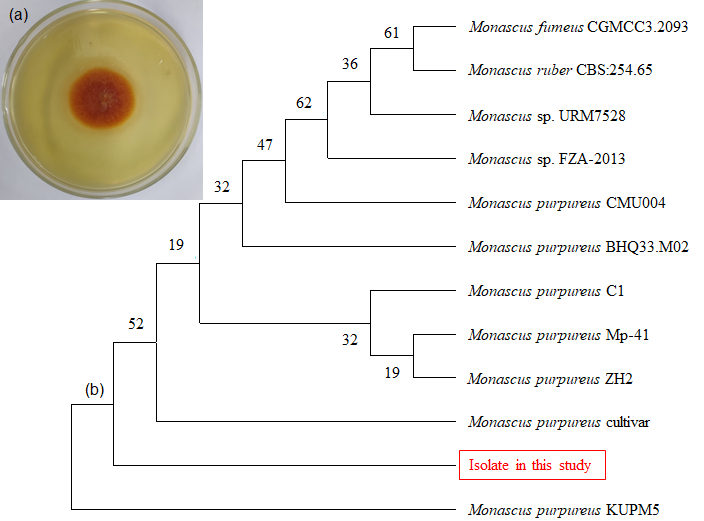


**Figure S1** Genetic evolutionary analysis for the isolated fungus strain. **a** The colony of the isolate. **b** Bootstrap consensus tree. The numbers on the branch indicated the node statistics.

**Figure S2** The effect of taurine on bioethanol fermentability for *Z. mobilis* ZM4. **a** Cell growth; **b** Glucose consumption; **c** Ethanol concentration; **d** Taurine concentration.

**Figure S3** The effect of taurine on itaconic acid fermentability for *A. terreus*. **a** Dry weight of mycelia; **b** Glucose consumption; **c** Xylose consumption; **d** Itaconic acid concentration; **e** Taurine concentration.

**Fig. S4** Transcriptional profiling for bioethanol and MonAzPs production of *M. purpureus* treated with taurine. **a** Validation of RNA-Seq data using qRT-PCR; **b** Homologous species distribution of the isolate in Non-Redundant Protein Sequence Database; **c** The DEGs; **d** The relative expression level of the most enriched significant DEGs; **e** GO analysis; **f** KEGG pathway analysis.

**Fig. S5** Taurine metabolism pathway and its gene expression level.
